# Moose in our neighborhood: Does perceived hunting risk have cascading effects on tree performance in vicinity of roads and houses?

**DOI:** 10.1002/ece3.8795

**Published:** 2022-04-03

**Authors:** Anne C. Mehlhoop, Bram Van Moorter, Christer M. Rolandsen, Dagmar Hagen, Aksel Granhus, Rune Eriksen, Thor Harald Ringsby, Erling J. Solberg

**Affiliations:** ^1^ Norwegian Institute for Nature Research (NINA) Trondheim Norway; ^2^ Department of Biology Centre of Biodiversity Dynamics Norwegian University of Science and Technology (NTNU) Trondheim Norway; ^3^ Norwegian Institute of Bioeconomy Research (NIBIO) Ås Norway

**Keywords:** *Alces alces*, browsing, human impacts, roads, tree recruitment, trophic cascade, ungulates

## Abstract

Like large carnivores, hunters both kill and scare ungulates, and thus might indirectly affect plant performance through trophic cascades. In this study, we hypothesized that intensive hunting and enduring fear of humans have caused moose and other forest ungulates to partly avoid areas near human infrastructure (perceived hunting risk), with positive cascading effects on recruitment of trees. Using data from the Norwegian forest inventory, we found decreasing browsing pressure and increasing tree recruitment in areas close to roads and houses, where ungulates are more likely to encounter humans. However, although browsing and recruitment were negatively related, reduced browsing was only responsible for a small proportion of the higher tree recruitment near human infrastructure. We suggest that the apparently weak cascading effect occurs because the recorded browsing pressure only partly reflects the long‐term browsing intensity close to humans. Accordingly, tree recruitment was also related to the density of small trees 5–10 years earlier, which was higher close to human infrastructure. Hence, if small tree density is a product of the browsing pressure in the past, the cascading effect is probably stronger than our estimates suggest. Reduced browsing near roads and houses is most in line with risk avoidance driven by fear of humans (behaviorally mediated), and not because of excessive hunting and local reduction in ungulate density (density mediated).

## Graphical abstract

Like large carnivores, hunters both kill and scare ungulates, and thus might indirectly affect plant performance through trophic cascades. We hypothesized that intensive hunting and enduring fear of humans have caused moose and other forest ungulates to partly avoid areas near human infrastructure, with positive cascading effects on recruitment of trees. We found decreasing browsing pressure and increasing tree recruitment in vicinity of human infrastructure, however, reduced browsing was only partly the reason for observed higher tree recruitment.

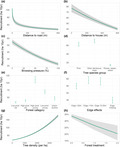

## 1 INTRODUCTION

Trophic cascades, where changes in abundance of one species affect another indirectly through the intermediate effects on one or more species, are common and observed in many ecosystems and food webs (Ford & Goheen, 2015; Pace et al., 1999; Ripple et al., 2016). In terrestrial systems, most trophic cascades involve a consumer limiting the abundance of its prey (top‐down), which then has consequences for the next lower trophic level, for example, primary producers (Pace et al., 1999; Ripple et al., 2016; Zhang et al., 2018). Such processes are often referred to as density mediated trophic cascades (DMTC, Ford & Goheen, 2015). Top‐down processes are, however, not limited to lethal effects but can also include changes in prey behavior in response to predation risk, also known as behaviorally mediated trophic cascades (BMTC, e.g., Schmitz et al., 1997). Due to fear of predation, wild ungulates may allocate more time and energy to vigilance, or they may avoid high‐risk habitats (Brown et al., 1999; Gaynor et al., 2019), which ultimately can lead to trophic cascades on plant performance and abiotic processes (Angelstam et al., 2017; Fortin et al., 2005; Kuijper et al., 2013; Ripple & Beschta, 2004).

During the last decades there has been a growing focus on potential trophic cascades on plant performance by the process of large carnivores consuming and scaring herbivores (Ford & Goheen, 2015). In most of Europe, however, large carnivores are scarce and in many areas they have just recently returned to their former ranges after decades of absence (Kuijper et al., 2016; Linnell et al., 2001). In contrast, high human activity, including hunting, is an important disturbance factor, and can lead to both numerical and antipredator responses in wild ungulates (Ciuti et al., 2012; Ripple & Beschta, 2004; Spitz et al., 2019). In Norway, the combined harvest of moose (*Alces alces*), red deer (*Cervus elaphus*), and roe deer (*Capreolus capreolus*) is almost 18 times higher than natural predation (Solberg et al., 2003), and a substantial number of wild ungulates are also killed by traffic. Trophic cascades on plant performance are, therefore, more likely to occur because of human disturbance (hunting, traffic) than large carnivore predation, but to what extent these are behaviorally or density mediated is less clear.

While DMTCs require a numerical reduction of herbivores caused by predation, BMTCs only require an antipredator response of herbivores to the risk of predation (Schmitz et al., 1997). Still, BMTCs are suggested to have a stronger effect on plant performance than DMTCs, particularly when BMTCs arise from risk‐averse habitat selection (Ford & Goheen, 2015; Preisser et al., 2005). Plants may for instance perform better in the vicinity of humans if herbivores find such areas riskier and, therefore, prefer to feed in safer areas, where the browsing pressure will consequently increase. This effect may be enhanced if the perception of risk extends beyond the hunting season (i.e., perceived risk of predation, Creel & Christianson, 2008; Frid & Dill, 2002) and involves humans in general and not only hunters. Indeed, even though ungulates may be hunted over large areas and not exclusively close to human settlements and roads, we hypothesize that it is merely the association between humans and the perceived risk of being killed that will shape the behavior of a risk‐averse ungulate.

In Norway, areas close to human infrastructure and settlements have experienced extensive woody plant encroachment during the last decades, often explained by the decline in the number of free‐ranging livestock (cattle and sheep) that peaked in the mid‐20th century (Aune & Hovstad, 2018; Speed et al., 2019). Coinciding with this, the abundance of wild herbivores (moose, red deer, and roe deer) has increased and the level of browsing by wild herbivores is now compensating for the decline in the former high level of livestock grazing in forest areas (Speed et al., 2019). Hence, rather than being due to a general decline in grazing and browsing, an alternative explanation for the woody plant encroachment could be that wild ungulates are not utilizing these areas to the same extent as livestock did in the past. Indeed, while livestock are habituated to human activity, regularly harvested ungulates may for good reasons fear humans and avoid such areas. Hypothetically, this may result in a trophic cascade on plant performance similar to what is observed when predators scare (BMTC) herbivores (Benhaiem et al., 2008; Bonnot et al., 2013; Ciuti et al., 2012; Creel & Christianson, 2008; Eccard & Liesenjohann, 2014; Ford et al., 2014; Kuijper et al., 2016; Ripple & Beschta, 2004; Thaker et al., 2011).

Here, we addressed this hypothesis (BMTC) by testing if variation in distance from human activities affects the variation in browsing pressure and plant performance. For this, we used nationwide data on browsing pressure, browse tree density, tree recruitment (out of browsing range), and other forest characteristics sampled on permanent plots by the National Forest Inventory (Norwegian Institute of Bioeconomy Research, 2019). We tested the hypothesis following the approach suggested by Ford and Goheen (2015): First, we examined if the predator (humans) affects wild herbivore behavior (BMTC) by testing the interaction between browsing pressure and distance to human settlements and infrastructure. As we assume that moose and deer perceive a higher predation risk in the vicinity of humans, we predicted (1) less browsing closer to houses and roads. Second, we examined if herbivory suppresses plant performance by testing if varying browsing pressure affects the recruitment of tree species preferred by moose and deer. We predicted (2) lower tree recruitment where the browsing pressure is high. Third, we examined if human activity indirectly facilitates tree recruitment by testing the relationship between tree recruitment and distance to humans, while controlling for browsing pressure. If human presence is generating a trophic cascade, we predicted (3) no (or weaker) relationship between tree recruitment and distance to houses and roads as all (or part of) the relevant variation in tree recruitment would be explained by variation in browsing pressure. We controlled for variation in forest productivity and forest structure in all analyses and discuss various mechanisms that can explain the pattern observed.

## 2 METHODS

### 2.1 Study area

**FIGURE 1:**
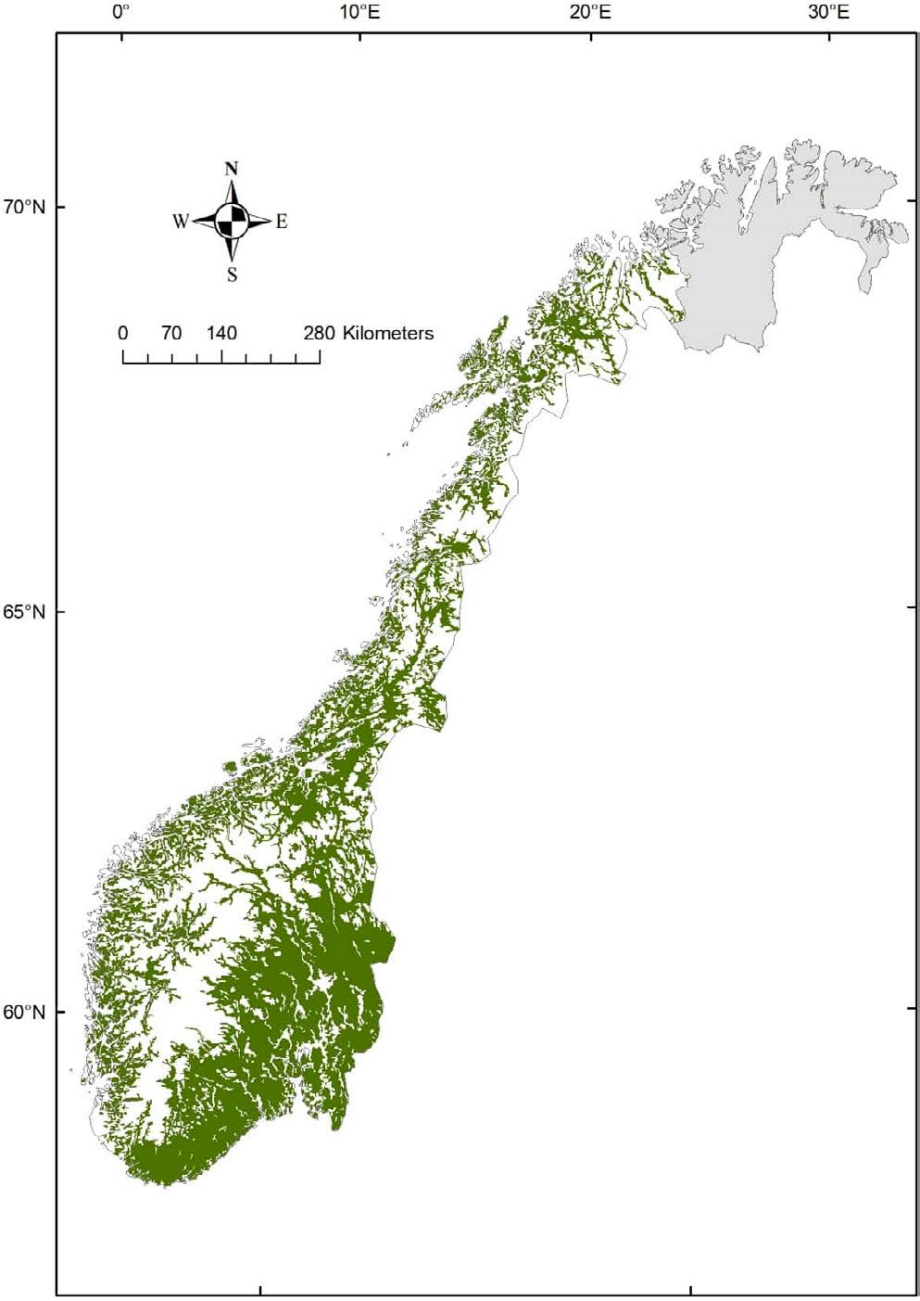
The study area covers all forested areas of Norway (green), except for the region of Finnmark (gray). The data on forest cover was retrieved from a Norwegian land cover map with a scale of 1:50,000 (AR50, Heggem et al., 2019)

The study area covers the forested area of mainland Norway (up to 1120 masl in altitude), except for the region of Finnmark in the north (Figure 1). Finnmark has low densities of wild cervids (mainly moose) and has only recently been included in the National Forest Inventory. The study area covers approximately 121 000 km^2^ (i.e., productive forest, unproductive forest, other wooded land; Svensson et al., 2021).

Most of the study area is within the boreal vegetation zone, with a small part entering the nemoral vegetation zone in the south (Moen, 1999). Forests are dominated by Norway spruce (*Picea abies*), Scots pine (*Pinus sylvestris*), and birches (downy birch *Betula pubescens*, silver birch *Betula pendula*), while grey alder (*Alnus incana*), aspen (*Populus tremula*), rowan (*Sorbus aucuparia*), and goat willow (*Salix caprea*) are found at lower densities. In the southern lowland areas, oak (*Quercus robur* and *Quercus petraea*), Norway maple (*Acer platanoides*), ash (*Fraxinus excelsior*), lime (*Tilia platyphyllos*), elm (*Ulmus glabra*), and beech (*Fagus sylvatica*) are also present. Downy birch is the main tree species growing in alpine and arctic woodland and may extend to 400 meters above the coniferous tree line (Bakkestuen et al., 2008; Moen, 1999).

Norway is diverse with regard to climate, with cold winters (average −10 to −4°C), mild summers (average 10 to 16°C) and relatively dry conditions (average 300–1000 mm) inland, and milder winters (average −3 to 4°C) and more humid conditions (average 500–3200 mm) near the coast (The Norwegian Meteorological Institute, 2020). At the coast of northern Norway, summers can be rather cool (average 8 to 10°C). Snow cover may last for 8 months to a few days each year, depending on latitude, altitude, and proximity to the sea (Moen, 1999).

### 2.2 Vegetation data and human activity

All vegetation data were collected by the National Forest Inventory (Breidenbach et al., 2020). The NFI have collected data on Norwegian forests through 11 inventory cycles since 1919 (the 12th cycle is ongoing). Since the 6th cycle (start in 1986) data have been sampled in permanent study plots, each 250 m^2^. The study plots are systematically distributed on a 3 × 3 km grid (one plot per grid cell) over the entire country below the coniferous tree line and on a 3 × 9 km grid above the coniferous tree line. One fifth of all permanent plots are surveyed each summer during a 5‐year cycle.

Variation in browsing pressure was analyzed based on data from the 9th cycle (2005–2009; *n* = 11,561), which was the first cycle in which data on browsing pressure were sampled. Browsing pressure was calculated as the proportion of twigs browsed of all available browsed and unbrowsed twigs on browse trees on the entire study plot, independent of when the twigs were browsed (i.e., during last or previous years). Browse trees are trees within 0.5–3.0 m height in the tree species groups: Scots pine, RAW‐trees (rowan, aspen, and willow pooled), and other deciduous trees pooled. The latter group is dominated by downy birch and to a lesser extent grey alder.

Variation in tree recruitment was analyzed based on data from the 10th and 11th cycle (2010–2014 and 2015–2019, respectively, *n* = 10,547). Recruits were defined as individual trees of Scots pine, RAW‐trees, and other deciduous trees that had grown into a diameter at breast height (DBH) of ≥50 mm since the 9th cycle (i.e., ingrowth trees). Above this diameter the tree crown is usually well above the browsing range for moose and other deer and thus the density of ingrowth trees may be used as a measure of tree recruitment.

From the NFI data, we also generated four control variables that are likely to affect browsing pressure or tree recruitment, and that may covary with distance to houses and roads (Table 1). First, based on the forest productivity (H_40_ site index system, Appendix S1) and forest development stage (maturity class 1–5, Appendix S1), we generated a variable called *forest category* with five levels: (1) low‐productive old forest, (2) high‐productive old forest, (3) low‐productive young forest, (4) high0productive young forest, and (5) unproductive forest. Young and old forests are stands at forest stages 1–2 and 3–5, respectively. Forest categories are likely to affect both tree recruitment and browsing pressure and because forest productivity tends to decrease with distance to humans, forest category needs to be controlled for in the models (Table 1).

**TABLE 1:** Focus and control variables included in the models and predicted effects

Independent variable	Description	Dependent variable	Inclusion explanation	Predicted effects
Distance to house	Proxy for human disturbance	Browsing pressure	Focus variable	Positive effect on browsing pressure and cascading negative effect on tree recruitment
		Tree recruitment		
Distance to road	Proxy for human disturbance	Browsing pressure	Focus variable	Positive effect on browsing pressure and cascading negative effect on tree recruitment
		Tree recruitment		
Browsing pressure	Proportion twigs browsed on browse trees	Tree recruitment	Focus variable	Lower recruitment at high browsing pressure
Browse tree species group	Factor with three levels: Scots pine; RAW‐trees (rowan, aspen, willow); other deciduous trees	Browsing pressure	Control variable. Covary with distance to humans (Appendix S7–S8, S10)	Higher browsing pressure on RAW‐trees than Scots pine and other deciduous trees
		Tree recruitment		Lower recruitment of RAW‐trees than Scots pine and other deciduous trees because of higher browsing pressure on RAW‐trees
Forest category	Factor with five levels: Old, high productive; young, high productive; old, low productive; young, low productive; unproductive	Browsing pressure	Control variable. Affects recruitment and browsing pressure. Covary with distance to humans (Appendix S7–S8, S10)	Higher browsing pressure and tree recruitment in young and more productive forest stands
		Tree recruitment		
Browse tree density	Number of browse trees (0.5–3.0 m tall) per ha	Browsing pressure	Control variable. Affects browsing pressure and tree recruitment. Covary with distance to humans (Appendix S7–S8, S10)	Higher browsing pressure and tree recruitment in stands with high tree density
		Tree recruitment		
Edge effect	Factor with four levels: Edge >20 m from plot; edge 10–20 m from plot; edge <10 m from plot; edge information missing	Browsing pressure	Control variable. Affect access to light (recruitment) and exposure to predators (browsing pressure). Covary with distance to humans (Appendix S7–S8, S10)	Higher browsing pressure farther from edges
		Tree recruitment		Higher tree recruitment closer to edges
Forest treatment	Weed control and precommercial thinning	Tree recruitment	Control variable. Likely to affect tree recruitment.	Lower tree recruitment in stands with more forest treatments

Second, based on data on edge type and distance to edge from the study plot, we generated a variable called *edge effect*. Forest edges are associated with light availability, which can affect plant growth and thus tree recruitment, and ungulates may also browse less intense close to edges to avoid predators (Table 1). Edges were only recorded for plots in productive forests and were in most cases an edge toward land cover types with more light (e.g., edge toward river, lake, road, farmland, young forest stand). The edge effect was categorized to 4 levels: (1) edge >20 m from plot (i.e., no edge effect), (2) edge 10–20 m from plot (slight edge effect), (3) edge within 10 m from plot (strong edge effect), and (4) no edge information. Level 4 includes all plots in unproductive forests. We did not distinguish between edge types in the analyses, except for edges toward older forest stands, which we characterized as no edge effect (level 4).

Third, we created a variable called *forest treatment* to control for the potential effect of weed control and precommercial thinning on tree recruitment. Such treatments are often conducted to improve the growth conditions for commercially important trees (Norway spruce or Scots pine) and may have removed tree recruits that otherwise would have been recorded in the 10th or 11th cycle. Plots that were not affected by forest treatment were given the value 0, whereas plot with one or both treatments were given the value 1 or 2, respectively.

The fourth control variable was *browse tree density* on sample plots. This is the density browse trees, on which browsing pressure was recorded (see above), and is measured as the number of trees per ha. Browse tree density was positively associated with browsing pressure and tree recruitment and was higher closer to human settlements and infrastructure (Table 1).

Distances to roads and houses were used as proxies of human activity and were measured as distance from study plot to closest road or house. Houses include buildings used by humans for permanent living or other daily use (e.g., factories, farm buildings). Private cabins or tourist cabins are not included as these are mainly used during holidays and not associated with intensive human activity. Roads include all private, municipality, county, and regional roads, as well as national highways. Forest roads were not included, because they are not used on daily basis, are often closed due to snow during winter, and thus are not likely to be associated with intensive human activity.

### 2.3 Cervid species

Browsing in the study area is mainly inflicted by moose (*Alces alces*), roe deer (*Capreolus capreolus*), and red deer (*Cervus elaphus*), which are the main wild forest‐dwelling cervids in Norway (reindeer, *Rangifer tarandus*, rarely use the same area, and is more of a grazer than a browser). Since moose is by far the most dominant in terms of biomass (e.g., Speed et al., 2019), we assume that most of the browsing pressure was inflicted by moose. Forests and woodlands constitute the main moose habitat in Norway, and moose are present in most of the country except for parts of western Norway (Speed et al., 2019). The second most dominant species is the red deer, which is mainly found in western and central Norway (Speed et al., 2019). Moose prefer to browse on rowan, aspen, and willow (RAW‐trees), followed by Scots pine and birches (Månsson et al., 2007; Wam & Hjeljord, 2010). Red deer are mixed feeders and feed more on forbs, dwarf bushes (e.g., *Vaccinium myrtillus*) and grasses compared to moose (Mysterud, 2000). Roe deer are mainly present in southern and central parts of the country, and like moose they feed on dwarf bushes and buds of deciduous trees during winter (Mysterud, 2000). Roe deer are probably more abundant than moose or red deer, but because of their smaller size (10% of the biomass of a moose), their impact on the vegetation is substantially lower.

### 2.4 Statistical analyses and predictions

Because of “zero‐inflated” data, we used hurdle models (Zeileis et al., 2008) to analyze the impact of human activity (i.e., distance to houses and roads) on browsing pressure and tree recruitment. Hurdle models are two‐component models, where one component (or model) is a binary regression model for modeling the zeros, and the other component being a Poisson‐ or negative binomial regression (Bolker, 2019; Zeileis et al., 2008). Each hurdle model consisted of a binary generalized linear mixed model (GLMM model, using lme4 package; Bates et al., 2015) and a zero‐truncated component (Beta regression for browsing data and Poisson for recruitment data, using glmmTMB package; Bolker, 2019; Brooks et al., 2017).

We obtained the predicted browsing pressure from the two components of the hurdle model, by multiplying the predicted probability of browsing (binary model) with the predicted proportion of browsing, given that browsing occurred (zero‐truncated model). The same procedure was applied to predict the recruitment of trees (Appendix S2 and S3). We used bootstrapping on the beta‐estimates to obtain confidence intervals (package boot; Canty & Ripley, 2017). All processing and analyses were done in R (version 3.5.2, R Core Team, 2018), using RStudio (version 1.1.456), and figures were produced using the packages ggplot2 (Wickham, 2016) and sjPlot (Lüdecke, 2018).

To analyze the nonlinear relationship between browsing pressure or tree recruitment and distance to house and road, we tried models with both log‐transformed distance and threshold distance and used AIC model selection (Burnham & Anderson, 2003) to determine which transformation worked best. We first used AIC to determine the best threshold distance above which infrastructure no longer affected browsing pressure or recruitment (i.e., zone of human influence) by contrasting full models with different threshold distances. Thus, in practice, we created a new distance variable where all distances above the threshold were set to the threshold distance (e.g., if threshold distance is 200 m, larger distances were set to 200 m), and then fitted a linear relationship with this new variable. Models including distance thresholds always performed better in modeling the effect of distance to house (Appendix S4: Tables S1 and S2) and distance to road on browsing pressure. However, in the actual model selection for the effect of distance to road, we encountered convergence issues for the models with a threshold. Hence, we decided to use a log‐relationship to model the effect of distance to road on browsing pressure (Appendix S5: Tables S1 and S2). Models including distance thresholds always performed better in modeling the effect of distance to house on tree recruitment (Appendix S4: Tables S3 and S4), whereas log‐transformation performed better in modeling the effect of distance to road (Appendix S5: Tables S3 and S4). If the threshold value differed between the two hurdle components, we used the best compromise (Appendix S5: Tables S3 and S4). Note that houses nearly always have access to roads, whereas roads are not always associated with houses. Distances to houses and roads were, therefore, positively correlated (*r* = .65, Appendix S6: Figure S1, Appendix S7: Figure S1).

In the GLMMs we examined the variation in browsing pressure and tree recruitment in relation to distance to houses, distance to roads, forest category, tree species group, edge effect, browse tree density, and, in the recruitment model only, browsing pressure and forest treatment (Table 1). To account for differences in other spatial factors (e.g., moose and deer density, climate), we added municipality as a random factor in all models and made a spatial‐autocorrelation variable (ac) (using autocovariate; Dormann et al., 2007). In preliminary models we also included slope and altitude as proxies of local moose density (i.e., within municipality) as moose may have problems utilizing steep terrain and avoid deep snow at higher altitudes. Both had the expected effect, but as their inclusion did not affect the impact of the focus variables (distance to houses and roads, browsing pressure), they were left out in order to simplify the models (Appendix S8).

In the full browsing pressure models, we included the main effects of all independent variables, as well as the effects of the two‐way interactions forest category × distance to house and forest category × distance to road. Similarly, we included all main effects in the full recruitment models. We also tested the two‐way interactions forest category × browsing pressure, forest category × distance to house, forest category × distance to road, and tree species group × browsing pressure. However, as the interactions did not substantially influence the outcome and subsequent analyses (potential cascading effect, see below) became too complicated, we decided not to include them further. In the browsing model, we predicted a stronger positive effect of distance to houses and roads in young compared to old forests because moose and deer are more visually exposed in open young forests, and thus are expected to be more wary when they feed in such habitats close to humans. For the same reasons, we predicted a stronger negative effect of distance to houses and roads on tree recruitment in young compared to old forests. Likewise, we predicted stronger effect of browsing on tree recruitment in old compared to young forests, because of less light and poorer growth conditions for understory trees in old forests, and stronger effects of browsing on the recruitment of RAW‐trees, as these species tend to experience substantially higher browsing pressure than other species.

For model selection (Appendix S8: Tables S1–S4) we used stepwise regression (a combination of backward elimination and forward selection), and AIC model selection (Burnham & Anderson, 2003). We selected the models with the lowest AIC as the best fitting model and show all models with ΔAIC ≤2 from the candidate models. However, if the second‐best model had ΔAIC ≥2, we also show that model (Appendix S8: Tables S1–S4).

To obtain the potential cascading effect of human on tree recruitment, we contrasted the estimated effect (slope) of distance to house and road from the best recruitment models (both hurdle components) with their estimated effects in the same model without browsing pressure included. If human avoidance has a cascading effect, we expected (1) decreasing browsing pressure toward roads and houses, (2) increasing tree recruitment toward roads and houses, and (3) no, or substantially less, effect of distance to houses and roads in the best recruitment model than in the same model excluding browsing pressure.

## 3 RESULTS

### 3.1 Data distribution

The browse trees in the study plots were dominated by deciduous trees (other than RAW‐trees, mainly birch), followed by RAW‐trees and Scots pine, and more plots were in low productive and unproductive forests compared to high‐productive forests (Appendix S9: Figure S1). The browsing pressure ranged from 0 to 99% (mean 25.9 SE ± 0.22), and the number of recruited trees (≥50 mm DBH) 5–10 years later ranged from 0 to 2952 individuals per ha (mean 48.6 SE ± 1.2).

The correlations between explanatory variables at the plot level were low (<0.3, except between distance to roads and houses, Methods, Appendix S6–S7), but several variables changed with distance to houses and roads. Study plots were on average located further from an edge as the distance to houses increased (Appendix S10: Figures S1 and S2), and, over the same gradient, forest productivity and browse tree density decreased (Appendix S10: Figures S1 and S2).

### 3.2 Browsing pressure

**FIGURE 2:**
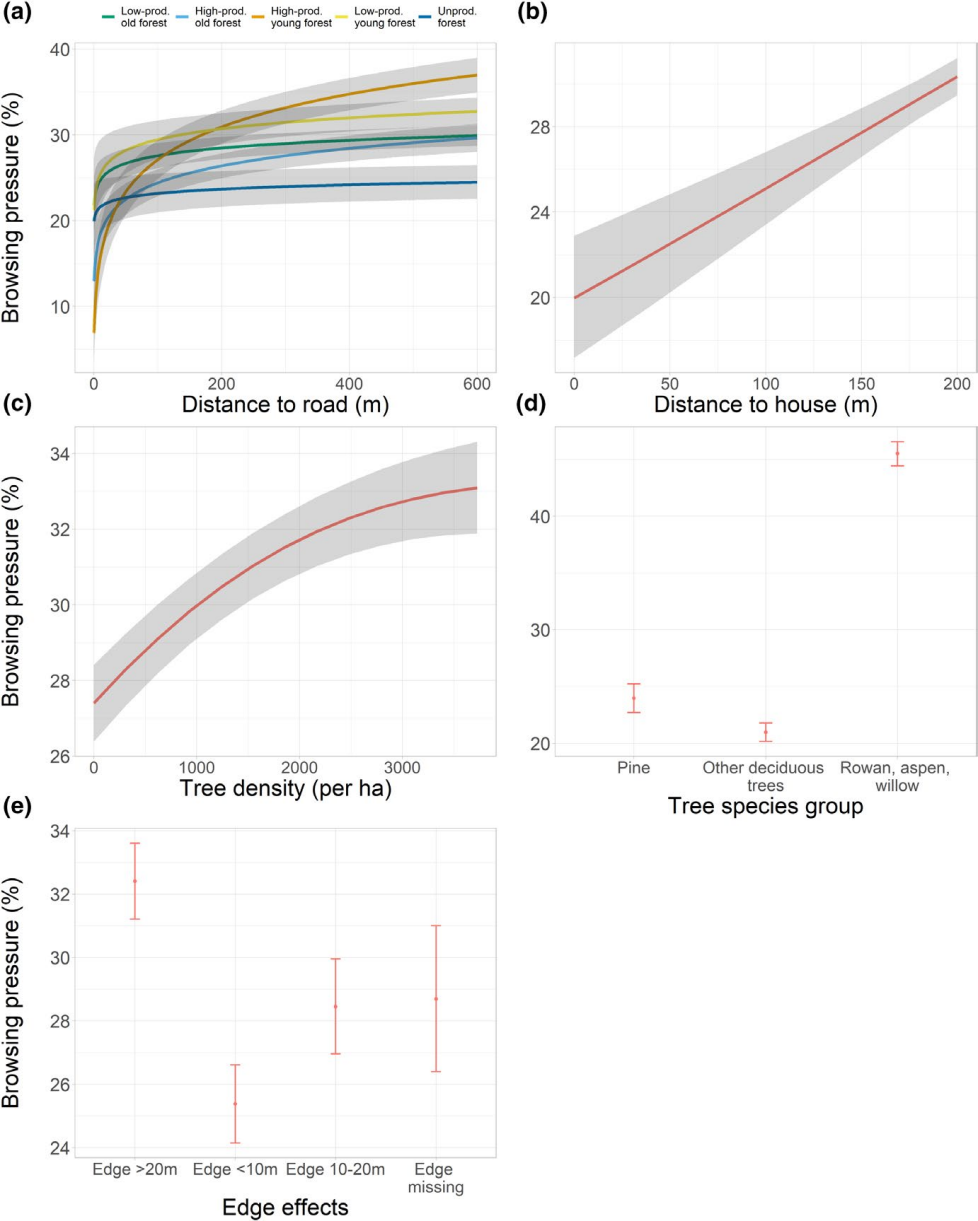
Predicted effects on browsing pressure (response variable) of (a) distance to road and forest category, (b) distance to house, (c) tree density, (d) tree species group, and (e) edge effect. Ribbons or error bars show bootstrapped 95% confidence intervals. For further information, see Methods

The model that best explained the variation in browsing pressure included the main effect of all explanatory variables, as well as the two‐way interaction forest category × distance to road (Figure 2, Appendix S11: Tables S1 and S2, Appendix S12: Figures S1 and S2). The browsing pressure increased with increasing distance to houses and roads, and more so in young than old and unproductive forests (Figure 2a). Model selection indicated that browsing increased up to 200 meters from houses (i.e., the threshold distance; Figure 2b), and most of the road effect on browsing pressure was found within the same range (the zone of human influence, Figure 2a). Browsing pressure increased from about 20% to 30% as the distance to house increased (Figure 2b).

The browsing pressure was higher on RAW‐trees (ca. 48%) than on Scots pine (ca. 24%) and other deciduous trees (ca. 22%; Figure 2d) and was on average highest in high‐productive young forests (ca. 30%) and lowest in unproductive forests (ca. 20%; Figure 2a). The browsing pressure also increased with increasing distance to edge (Figure 2e) and was higher in plots with high compared to low tree density (Figure 2c).

### 3.3 Recruitment of trees

**FIGURE 3:**
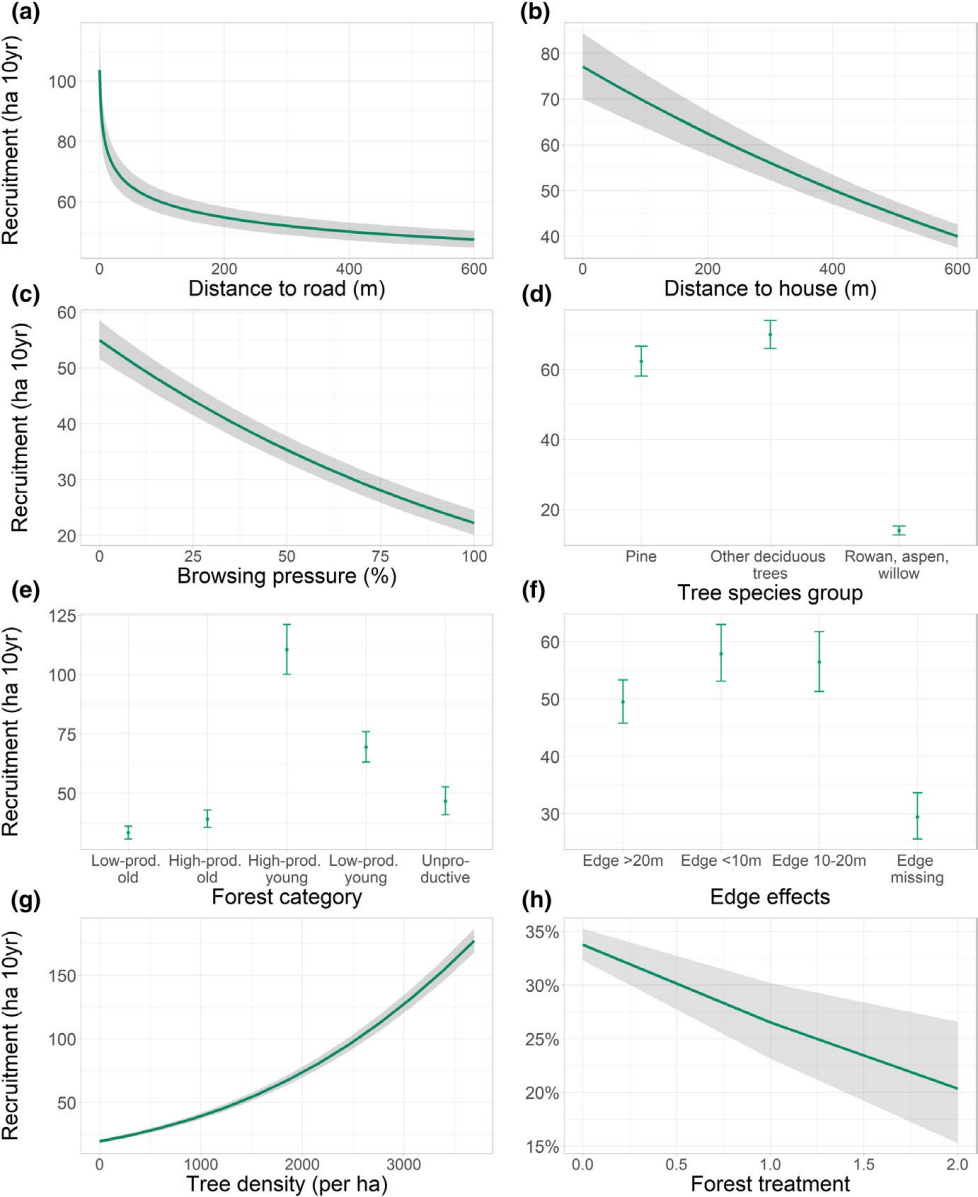
Predicted effects on recruitment of browse trees (number of recruited trees per ha over 10 years) of (a) distance to road, (b) distance to house, (c) browsing pressure, (d) tree species group, (e) forest category, (f) edge effect, (g) tree density, and (h) forest treatment (predicted probability of recruitment of browse trees, included in binary model only). Ribbons or error bars show bootstrapped 95% confidence intervals. For further information, see Methods

The best recruitment model included all explanatory variables (Figure 3, Appendix S11: Tables S3 and S4, Appendix S12: Figures S3 and S4) and suggested that tree recruitment decreased to a threshold distance of 600 meters (Appendix S11: Tables S3–S4, Appendix S12: Figures S3 and S4). Recruitment was negatively affected by distance to roads and houses, even with browsing pressure included in the model (Figure 3a–c). Removing browsing pressure from the best model, the estimated effect (slope) of distance to house increased by about 3% in the binary component (Appendix S13: Tables S1 and S2) and 10% in the zero‐truncated component (Appendix S13: Tables S3 and S4). For distance to roads the estimated effect increased with 7% in the binary component (Appendix S13: Tables S1 and S2) and 12% in the zero‐truncated component (Appendix S13: Tables S3 and S4). Hence, the decline in browsing pressure was only to a limited extent responsible for the increase in tree recruitment closer to roads and houses, which was less than expected based on prediction 3 (Introduction). Indeed, as a substantial part of the decline in tree recruitment occurred at distances beyond 200 meters from houses and roads (i.e., the zone of human influence for browsing pressure, Figure 3b), browsing pressure is unlikely to be the sole factor explaining the negative relationship between tree recruitment and distance to human infrastructure.

Recruitment also increased with increasing browse tree density and decreasing forest treatment, and varied with tree species group, forest category, and distance to edge (Figure 3d–h). The fact that control variables were retained in the best model suggests that these covariates affect recruitment partly independent of their effects on browsing pressure. However, the direction of effect was as expected based on their predicted indirect effect through browsing pressure. Particularly striking was the substantial direct effect of tree species group on tree recruitment as, for this variable, we mainly expected indirect effects working through browsing pressure (Table 1). The direct positive effect of tree density and negative effect of forest treatment on tree recruitment were both in line with expectations.

## 4 DISCUSSION

The present study has demonstrated that large, wild herbivores are browsing less close to human infrastructure, and that this has cascading effects on the recruitment of browse trees. We hypothesized that moose and deer are less prevalent near houses and roads because of higher perceived hunting risk, and that subsequent reduction in browsing leads to higher recruitment of preferred browse trees within the zone of human influence (cascading effect). As expected, we found lower browsing pressure in the vicinity of houses and roads (prediction 1, Introduction) and higher tree recruitment on study plots with lower browsing pressure (prediction 2, Introduction) (Figure 3). In addition, we found higher recruitment of trees closer to roads and houses (Figure 3), but this could only to a small extent be explained by reduced browsing (less support for prediction 3). Below we suggest two possible mechanisms that may explain the rather low cascading effect: (1) that we have not provided sufficiently statistical control for factors that improve tree recruitment close to human infrastructure and (2) that tree recruitment is also affected by risk avoidance in the past. Indeed, as variation in tree recruitment was also affected by browse tree density 5–10 years earlier, which in turn increased toward human infrastructure, we suspect that the current high tree recruitment may also be a product of less browsing prior to the time when browsing pressure was recorded (9th cycle).

Many ungulate species alter their habitat use in response to predation risk by carnivores, and a similar behavior may appear as a response to hunting risk (Bonnot et al., 2013; Ciuti et al., 2012; Cleveland et al., 2012; Lone et al., 2017; Morgantini & Hudson, 1985; Proffitt et al., 2009). In our study, we analyzed the variation in browsing pressure and not behavior per se, and we, therefore, cannot conclusively claim that reduced browsing is due to risk avoidance. It could for instance be that moose (and other ungulates) that live near roads and houses are more likely to be killed by hunters (because of better hunter access) or by cars, which will generate a gradient of increasing density and browsing away from human infrastructure (i.e., a density mediated response). Indeed, as density and behaviorally mediated responses often occur concurrently (e.g., Ford & Goheen, 2015), it is not straight forward to quantitatively separate their relative importance.

For several reasons, we believe that density‐mediated reduction in browsing pressure is the least likely explanation for the pattern observed. First, in Norway, moose hunting is conducted within relatively small hunting fields (on average approximately 20 km^2^ of forests and bogs, C. M. Rolandsen unpublished data) and the number of hunting permits is in most cases scaled to the area of forest land available. This restricts hunters from killing more ungulates in hunting fields that are close to human infrastructure, even if such hunting fields are easier to access. Secondly, the influence zone was rather small as the browsing pressure increased only up to 200 m from houses and roads before it levelled off. This is far below the extent of a moose home range, and smaller than the distances moved by an average moose within a day (Van Moorter et al., 2013). If elevated hunting pressure (or traffic mortality) was the culprit, we would have expected a much wider and less abrupt influence zone. This assumption is also supported by several studies showing that radio‐collared moose spend less time in the vicinity of houses (Lykkja et al., 2009) and roads (Eldegard et al., 2012) and are less inclined to use open habitats during daytime (Bjørneraas et al., 2011).

So, why is it that forest ungulates perceive areas close to roads and houses as particularly risky? If the hunting risk is spatially unpredictable, as suggested above, wild ungulates should be vigilant in all forested areas during the hunting season (Creel et al., 2005), and not only close to humans. We believe the answer is found in how wild ungulates perceive predation risk (Frid & Dill, 2002), and how humans in general are distributed in the landscape. During the hunting season, wild ungulates regularly experience life‐threatening encounters with hunters and their dogs and may also be traumatized by losing a calf or accompanying conspecific. This may inflict a hunter‐induced fear with enduring effects on their subsequent reactions to humans in general. Accordingly, for a moose or deer, the risk of frequent and unpredictable encounters with humans is likely to trigger higher vigilance or avoidance of such areas even outside the hunting season (Lykkja et al., 2009).

As intensive browsing is likely to affect the growth and vitality of trees (Speed et al., 2013), we predicted a significant increase in the number of trees that were able to grow to heights above browsing range as the browsing pressure declined. In support of this prediction, we found a negative relationship between browsing pressure and tree recruitment and substantially more tree recruits in the vicinity of humans in all forest types. However, the higher recruitment closer to humans was not always found on plots with low browsing pressure. Indeed, as the zone of human influence was substantially lower for browsing pressure (about 200 meters) than for tree recruitment (600 meters), it is likely that at least part of the increase in tree recruitment close to humans is caused by something else than reduced browsing. In the best models, many covariates besides browsing pressure explained a substantial part of the variation in tree recruitment but could not fully explain this larger zone of human influence.

Another possibility is that measurement errors in browsing pressure have generated the unexpected result. Measurement error may reduce the effectiveness of statistically controlling for a mediating variable, which may result in spurious effects (Cole & Preacher, 2014). Specifically, in our analysis, the relatively strong relationship between distance to human infrastructure and tree recruitment after controlling for browsing pressure, could be a spurious one due to measurement errors in browsing pressure. For instance, if it is more difficult to correctly assess the browsing pressure when it is relatively high, compared to intermediate or low. This could explain the much smaller effect zone for browsing pressure compared to that for tree recruitment. Unfortunately, no estimates of measurement error are available for browsing pressure, but it is likely to be rather substantial as the monitoring of browsing pressure was altered in later inventories due to perceived poor reliability (A. Granhus, unpublished data). The mediating effect of browsing pressure on tree recruitment could, therefore, turn out to be stronger in future studies when more accurate estimates of browsing pressure are available.

When interpreting the results, we should also bear in mind that wild ungulates have probably avoided human neighborhoods for a long time, and that the conditions for improved tree recruitment may have accumulated over time. The fact that browse tree density had a positive effect on the number of tree recruits and also increased toward human infrastructure (Appendix S14: Table S1, Figure S1), may support such a notion. It is likely that the density of browse trees, at least in part, is affected by the number of reproducing trees in the previous generation, which distribution may in turn have been formed by varying degrees of risk avoidance in the past. Such a legacy effect could have been spurred by the tree encroachment that occurred after the reduction in free ranging livestock and before the subsequent increase in wild ungulate populations (late 1960s, see Introduction). In this period, the encroachment was likely to be stronger in the most intensively grazed areas closer to settlements, because wild ungulates were few and feared humans. Unfortunately, we have no long‐term data to test this hypothesis, but for lack of a better explanation, we cannot exclude that elevated hunting risk is also the causal factor behind the higher density of browse trees closer to human infrastructure.

Besides documenting a potential cascading effect of hunting risk on tree recruitment, our findings may have implications for biodiversity conservation and traffic safety. The higher tree recruitment closer to houses and roads suggests that such areas are now providing a sanctuary for vulnerable tree species in Norwegian forests. Rowan, aspen, and sallow are not only important trees in the forest ecosystem (Myking et al., 2011, 2013) but are also the species most preferred by moose (e.g., Månsson et al., 2007). In areas with high moose density, they are, therefore, struggling to recruit (Kolstad et al., 2018), except in inaccessible terrain, and—as suggested by our results—in areas with high perceived hunting risk. As such, hunting‐induced fear can, unintentionally, have acted as a management tool to divert ungulates from roads and residential areas (i.e., “hunting for fear,” Cromsigt et al., 2013), where their presence is undesired also for safety reasons. Each year more than 10,000 moose and deer are hit by cars and trains in Norway, of which most are killed, and many are injured (C. M. Rolandsen, unpublished data). However, we suspect that the amount of moose and deer involved in a traffic accident would be much higher if the zone of human influence was used in accordance with the availability of food.

## CONFLICT OF INTEREST

The authors declare that there have no conflicts of interest.

## AUTHOR CONTRIBUTIONS

**Anne Catriona Mehlhoop:** Conceptualization (equal); Data curation (lead); Formal analysis (lead); Writing – original draft (lead); Writing – review & editing (lead). **Bram Van Moorter:** Conceptualization (equal); Data curation (supporting); Formal analysis (equal); Supervision (equal); Writing – original draft (supporting); Writing – review & editing (supporting). **Christer Rolandsen:** Conceptualization (equal); Data curation (supporting); Formal analysis (supporting); Supervision (equal); Writing – original draft (equal); Writing – review & editing (equal). **Dagmar Hagen:** Conceptualization (supporting); Supervision (equal); Writing – original draft (supporting); Writing – review & editing (supporting). **Aksel Granhus:** Data curation (lead); Writing – review & editing (supporting). **Rune Eriksen:** Data curation (lead); Writing – review & editing (supporting). **Thor‐Harald Ringsby:** Conceptualization (equal); Supervision (equal); Writing – original draft (equal); Writing – review & editing (equal). **Erling Solberg:** Conceptualization (lead); Data curation (equal); Formal analysis (supporting); Supervision (equal); Writing – original draft (equal); Writing – review & editing (equal).

## Supporting information

Supplementary Material

## Data Availability

The data that support the findings of this study are openly available in Dryad at https://doi.org/10.5061/dryad.xpnvx0khk.
