# Supplementary material for: Moose in our neighborhood: Does perceived hunting risk have cascading effects on tree performance in vicinity of roads and houses?
Source: Ecol Evol. 2022 Apr 3;12(4):e8795. doi: 10.1002/ece3.8795 (PMC8977646; doi:10.1002/ece3.8795)
Supplement: Supplementary file 1 — Supplementary Material [file ECE3-12-e8795-s001.docx]

Appendix S1

**Additional explanation of forest category**

The NFI (National Forest Inventory) broadly classifies stands to unproductive forest (< 1 m^3^ wood produced per ha and year) and productive forest (≥ 1 m^3^ wood produced per ha and year), of which the latter is divided into another eight productivity classes. Forest productivity is measured in H_40_ system, which refers to the height of Norway spruce or Scots pine at the age of 40 years at breast height. Categories in use are productivity class 6, 8, 11, 14, 17, 20, 23 and 26. Likewise, the NFI classifies productive forest stands into five development stages: 1 recently cleared; 2 rejuvenating stands; 3 and 4 young and old production stands (mainly for thinning); and 5 commercially mature stands. Stands may reach a given development stage at various ages, depending on the forest productivity (temperature, humidity and soil nutrients). Trees at stage 2 stands are typically below 10-12 meters and provide most feed for moose. However, they also provide moose with less cover than stands in higher cutting classes. Unproductive forests are seldom cut and are not classified into development stages.

Appendix S2

**Flow chart of the analysis.**


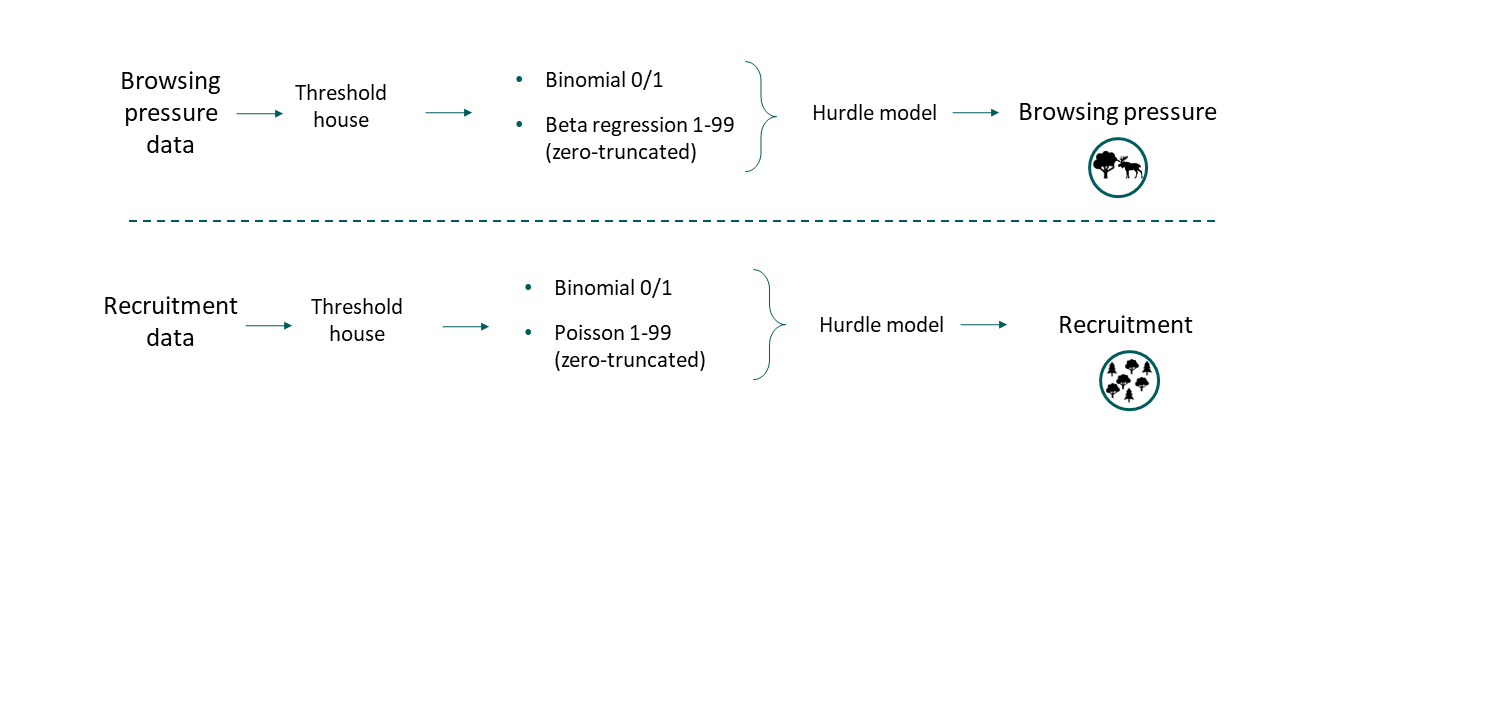


**Fig. S1. Flow chart of the analysis.** Each hurdle model consists of a binary and a zero-truncated component, which are combined to predict the total expected browsing pressure or recruitment, respectively. For further information on how we combined the models, see equations on page 3.

Appendix S3

**Equations used to combine the hurdle model components and calculate effects**

BP: browsing pressure

R: recruitment

$\beta\boldsymbol{x}$: the matrix of the covariates (except for the focal variable FV, or browsing pressure BP) multiplied by the regression coefficients

FV: is the vector with the focal variable

Browsing pressure:

${BP}^{01}=logit(\beta\boldsymbol{x}\boldsymbol{+}\boldsymbol{\beta}_{\boldsymbol{FV}}\boldsymbol{FV}$**)**

${BP}^{>0}=logit(\beta\boldsymbol{x}\boldsymbol{+}\boldsymbol{\beta}_{\boldsymbol{FV}}\boldsymbol{FV}$**)**

$$BP={BP}^{01}\times{BP}^{>0}$$

Recruitment:

$R^{01}=logit(\beta\boldsymbol{x+}\boldsymbol{\beta}_{\boldsymbol{BP}}\boldsymbol{BP+}\boldsymbol{\beta}_{\boldsymbol{FV}}\boldsymbol{FV}$**)**

$R^{>0}=logit(\beta\boldsymbol{x+}\boldsymbol{\beta}_{\boldsymbol{BP}}\boldsymbol{BP+}\boldsymbol{\beta}_{\boldsymbol{FV}}\boldsymbol{FV}$**)**

$$R=R^{01}\times R^{>0}$$

Appendix S4 Model selection threshold distance to house

**Browsing pressure as response variable**

AIC selection for threshold distance to house for the binary model component (Table S1) and the zero-truncated model component (Table S2) of the hurdle for browsing pressure. The only variable changing is distance to house, to determine the threshold when distance to house has no effect on browsing pressure. To test the different thresholds, all distances above the certain threshold were set to the threshold (e.g. threshold 200m, all distances > 200m were set to 200m). The models are shown in decreasing order with best fitting model at the top, and only models with ΔAICc ≤ 2 are included for better readability. However, if the model with second lowest ΔAIC had a ΔAIC ≥ 2, we show that model. Variables included in the model are noted with x, variables not included are noted with -.

**Table S1 Browsing, threshold house binary, total 8 models tested**

| Model | Distance to house (200m) | Distance to house (150m) | Distance to road | Forest category | Tree species group | Autocorrelation | Edge effect | Slope | Altitude | Tree density | Moose density | AIC | Delta AIC | AIC weight |
| --- | --- | --- | --- | --- | --- | --- | --- | --- | --- | --- | --- | --- | --- | --- |
| Mod7 | x | - | x | x | x | x | x | x | x | x | x | 21508.86 | 0 | 0.66 |
| Mod8 | - | x | x | x | x | x | x | x | x | x | x | 21510.71 | 1.84 | 0.26 |

**Table S2 Browsing, threshold house zero-truncated, total 8 models**

| Model | Distance to house (200m) | Distance to house (300m) | Distance to road | Forest category | Tree species group | Autocorrelation | Edge effect | Slope | Altitude | Tree density | Moose density | AICc | Delta AICc | AICc weight |
| --- | --- | --- | --- | --- | --- | --- | --- | --- | --- | --- | --- | --- | --- | --- |
| Mod7 | x | - | x | x | x | x | x | x | x | x | x | -9734.48 | 0 | 0.92 |
| Mod5 | - | x | x | x | x | x | x | x | x | x | x | -9728.16 | 6.32 | 0.04 |

🡪 Continued on page 5.

**Recruitment of trees as response variable**

AIC selection for threshold distance to house for the binary model component (Table S3) and the zero-truncated model component (Table S4) of the hurdle for recruitment. The only variable changing is distance to house, to determine the threshold when distance to house has no effect on recruitment. To test the different thresholds, all distances above the certain threshold were set to the threshold (e.g. threshold 200m, all distances > 200m were set to 200m). The models are shown in decreasing order with best fitting model at the top, and only models with ΔAICc ≤ 2 are included for better readability. However, if the model with second lowest ΔAIC had a ΔAIC ≥ 2, we show that model. Variables included in the model are noted with x, variables not included are noted with -.

**Table S3 Recruits, threshold house binary, total 9 models**

| Model | Distance to house (600m) | Distance to house (500m) | Distance to road | Forest category | Tree species group | Auto-  correlation | Edge effect | Slope | Altitude | Browsing pressure | Moose density | Forest treatment | Tree density | AIC | Delta AIC | AIC weight |
| --- | --- | --- | --- | --- | --- | --- | --- | --- | --- | --- | --- | --- | --- | --- | --- | --- |
| Mod2 | x | - | x | x | x | x | x | x | x | x | x | x | x | 21532.9 | 0 | 0.67 |
| Mod3 | - | x | x | x | x | x | x | x | x | x | x | x | x | 21534.54 | 1.64 | 0.29 |

**Table S4 Recruits, threshold house zero-truncated, total 8 models**

| Model | Distance to house (600m) | Distance to house (500m) | Distance to road | Forest category | Tree species group | Autocorrelation | Edge effect | Slope | Altitude | Browsing pressure | Moose density | Tree density | Forest treatment | AIC | Delta AIC | AIC weight |
| --- | --- | --- | --- | --- | --- | --- | --- | --- | --- | --- | --- | --- | --- | --- | --- | --- |
| Mod2 | x | - | x | x | x | x | x | x | x | x | x | x | x | 1017360 | 0 | 1 |
| Mod3 | - | x | x | x | x | x | x | x | x | x | x | x | x | 1017542 | 182.2 | 0 |

Appendix S5 Model selection threshold distance to roads and test log-relationship

**Browsing pressure as response variable**

AIC selection for threshold distance to roads for the binary model component (Table S1) and the zero-truncated model component (Table S2) of the hurdle for browsing pressure. The only variable changing is distance to roads, to determine the threshold when distance to roads has no effect on browsing pressure. To test the different thresholds, all distances above the certain threshold were set to the threshold (e.g. threshold 200m, all distances > 200m were set to 200m). We then tested if a log-relationship with distance to roads fitted better than the best fitting threshold model. The models are shown in decreasing order with best fitting model at the top, and only models with ΔAICc ≤ 2 are included for better readability. However, if the model with second lowest ΔAIC had a ΔAIC ≥ 2, we show that model. Variables included in the model are noted with x, variables not included are noted with -.

**Table S1 Browsing pressure, threshold road binary, total 10 models tested**

| Model | Distance to road (200m) | Distance to road (150m) | Distance to house (200m) | Forest category | Tree species group | Autocorrelation | Edge effect | Slope | Altitude | Tree density | Moose density | AIC | Delta AIC | AIC weight |
| --- | --- | --- | --- | --- | --- | --- | --- | --- | --- | --- | --- | --- | --- | --- |
| Mod8 | - | x | x | x | x | x | x | x | x | x | x | 21409.97 | 0 | 0.98 |
| Mod7 | x | - | x | x | x | x | x | x | x | x | x | 21417.86 | 7.89 | 0.02 |

**Table S2 Browsing pressure, threshold road zero-truncated, total 8 models**

| Model | Distance to road (200m) | Distance to road (150m) | Distance to house | Forest category | Tree species group | Autocorrelation | Edge effect | Slope | Altitude | Tree density | Moose density | AICc | Delta AICc | AICc weight |
| --- | --- | --- | --- | --- | --- | --- | --- | --- | --- | --- | --- | --- | --- | --- |
| Mod7 | x | - | x | x | x | x | x | x | x | x | x | -9780.7 | 0 | 0.82 |
| Mod8 | - | x | x | x | x | x | x | x | x | x | x | -9777.44 | 3.26 | 0.16 |

For both model components, the threshold fitted better, however, using a threshold for distance to roads caused massive convergence problems later in the model selection, which is why we chose to use a log relationship for distance to roads in all browsing pressure models.

🡪 Continued on page 7.

**Recruitment of trees as response variable**

AIC selection for threshold distance to roads for the binary model component (Table S3) and the zero-truncated model component (Table S5) of the hurdle for recruitment. The only variable changing is distance to roads, to determine the threshold when distance to roads has no effect on recruitment. To test the different thresholds, all distances above the certain threshold were set to the threshold (e.g. threshold 200m, all distances > 200m were set to 200m). We then tested if a log-relationship with distance to roads fitted better than the best fitting threshold model (Table S4, Table S6). The models are shown in decreasing order with best fitting model at the top, and only models with ΔAICc ≤ 2 are included for better readability. However, if the model with second lowest ΔAIC had a ΔAIC ≥ 2, we show that model. Variables included in the model are noted with x, variables not included are noted with -.

**Table S3 Recruits, threshold road binary, total 9 models**

| Model | Distance to road (500m) | Distance to road (400m) | Distance to road (350m) | Distance to house | Forest category | Tree species group | Autocorrelation | Edge effect | Slope | Altitude | Browsing pressure | Moose density | Forest treatment | Tree density | AIC | Delta AIC | AIC weight |
| --- | --- | --- | --- | --- | --- | --- | --- | --- | --- | --- | --- | --- | --- | --- | --- | --- | --- |
| Mod3 | x | - | - | x | x | x | x | x | x | x | x | x | x | x | 21523.16 | 0 | 0.36 |
| Mod4 | - | x | - | x | x | x | x | x | x | x | x | x | x | x | 21523.37 | 0.2 | 0.32 |
| Mod5 | - | - | x | x | x | x | x | x | x | x | x | x | x | x | 21525.46 | 2.3 | 0.11 |

**Table S4 Recruits, threshold or log-transformation road binary, total 3 models**

| Model | Distance to road (500m) | Distance to road (400m) | log Distance to road | Distance to house | Forest category | Tree species group | Autocorrelation | Edge effect | Slope | Altitude | Browsing pressure | Moose density | Forest treatment | Tree density | AIC | Delta AIC | AIC weight |
| --- | --- | --- | --- | --- | --- | --- | --- | --- | --- | --- | --- | --- | --- | --- | --- | --- | --- |
| Mod_log | - | - | x | - | x | x | x | x | x | x | x | x | x | x | 21511.59 | 0 | 0.62 |
| Mod3 | x | - | - | x | x | x | x | x | x | x | x | x | x | x | 21513.73 | 2.14 | 0.21 |
| Mod4 | - | x | - | x | x | x | x | x | x | x | x | x | x | x | 21514.2 | 2.61 | 0.17 |

🡪 Continued on page 8.

**Table S5 Recruits, threshold house zero-truncated, total 8 models**

| Model | Distance to road (600m) | Distance to road (150m) | Distance to house | Forest category | Tree species group | Autocorrelation | Edge effect | Slope | Altitude | Browsing pressure | Moose density | Tree density | Forest treatment | AIC | Delta AIC | AIC weight |
| --- | --- | --- | --- | --- | --- | --- | --- | --- | --- | --- | --- | --- | --- | --- | --- | --- |
| Mod8 | - | x | x | x | x | x | x | x | x | x | x | x | x | 1018667 | 0 | 1 |
| Mod2 | x | - | x | x | x | x | x | x | x | x | x | x | x | 1018734 | 67.37 | 0 |

**Table S6 Recruits, threshold or log-transformation road zero-truncated, total 2 models**

| Model | Distance to house (600m) | Distance to house (500m) | Distance to road | Forest category | Tree species group | Autocorrelation | Edge effect | Slope | Altitude | Browsing pressure | Moose density | Tree density | Forest treatment | AIC | Delta AIC | AIC weight |
| --- | --- | --- | --- | --- | --- | --- | --- | --- | --- | --- | --- | --- | --- | --- | --- | --- |
| Mod1 | - | x | x | x | x | x | x | x | x | x | x | x | x | 1016191 | 0 | 1 |
| Mod2 | x | - | x | x | x | x | x | x | x | x | x | x | x | 1016494 | 302.7 | 0 |

Appendix S6 Correlation plots browsing pressure data


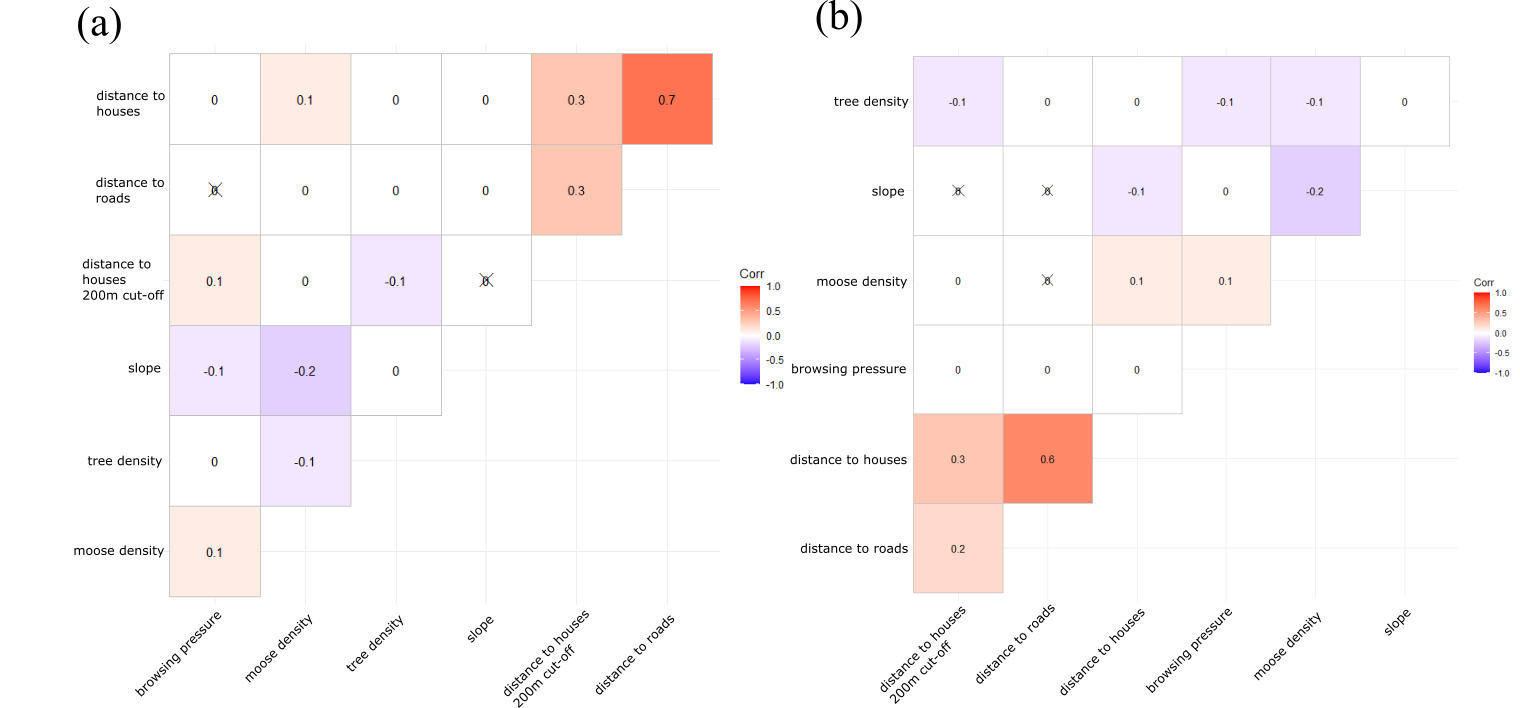


Figure S1: Correlation plots of the browsing pressure data. Panel (a) shows the correlation for the binary data and (b) for the zero-truncated data. Significant values are crossed out.

Appendix S7 Correlation plots recruitment data


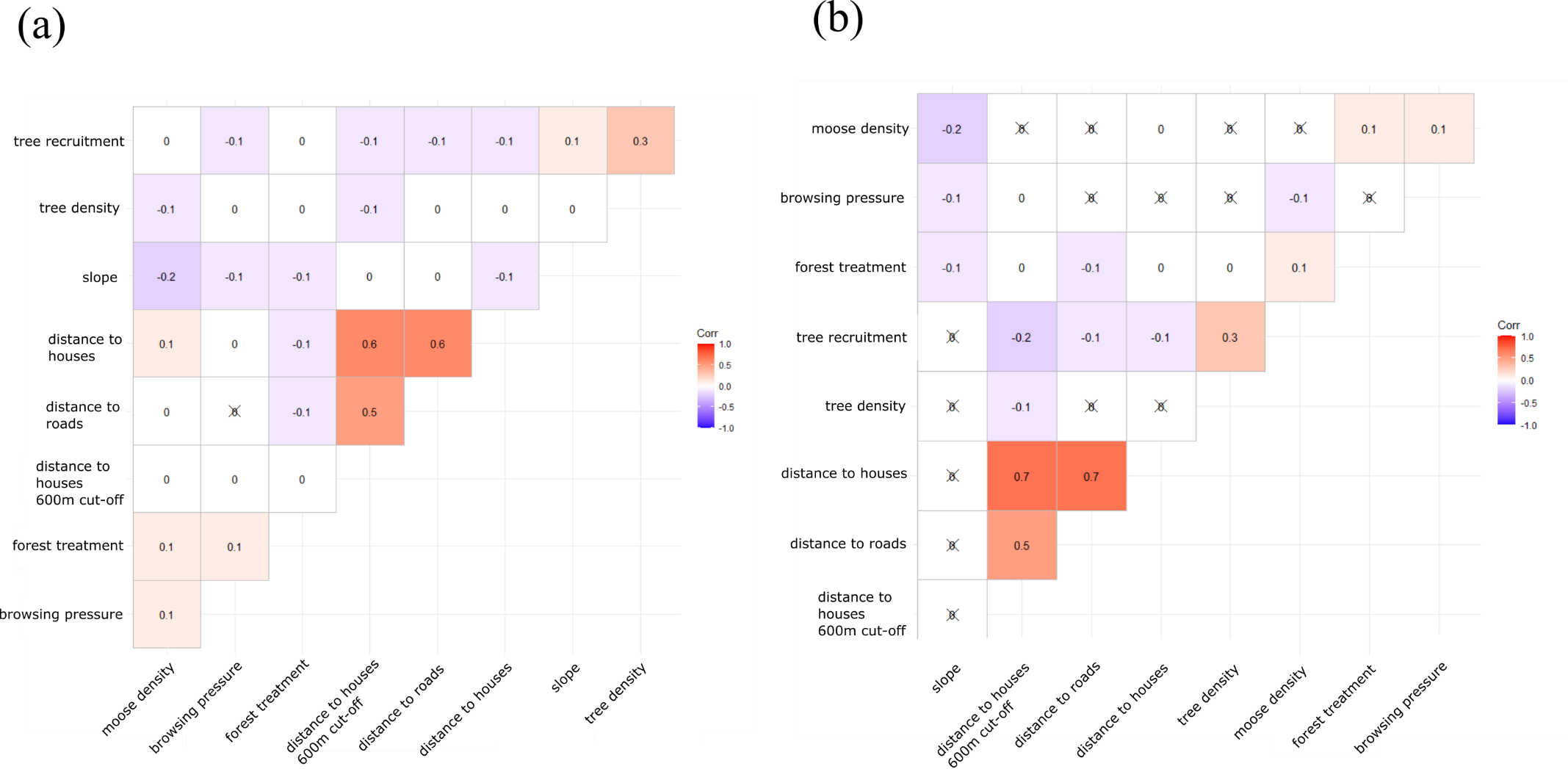


Figure S1: Correlation plots of the recruitment data. Panel (a) shows the correlation for the binary data and (b) for the zero-truncated data. Significant values are crossed out.

Appendix S8 Candidate models that received the highest support explaining the variation in browsing pressure and recruitment, and explanation on dropping explanatory variables

**Browsing pressure as response variable**

AIC selection for candidate models for the binary model component (Table S1) and the zero-truncated model component (Table S2) of the hurdle for browsing pressure. The models are shown in decreasing order with best fitting model at the top, and only models with ΔAICc ≤ 2 are included for better readability. However, if the model with second lowest ΔAIC had a ΔAIC ≥ 2, we show that model. Variables included in the model are noted with x, variables not included are noted with -.

**Table S1 Binary component of hurdle model, total 5 models tested**

| Model | Distance to house (200m) | log distance to road*Forest category | Distance to house (200m)*Forest category | Tree species group | Autocorrelation | Edge effect | Slope | Altitude | Tree density | Moose density | AIC | Delta AIC | AIC weight |
| --- | --- | --- | --- | --- | --- | --- | --- | --- | --- | --- | --- | --- | --- |
| Mod5 | x | x | - | x | x | x | x | x | x | x | 21451.17 | 0 | 0.62 |
| Mod4 | - | x | x | x | x | x | x | x | x | x | 21452.16 | 0.99 | 0.38 |

**Table S2 Zero-truncated component of hurdle model, total 5 models tested**

| Model | Distance to house (200m) | log distance to road*Forest category | Tree species group | Autocorrelation | Edge effect | Slope | Altitude | Tree density | Moose density | AIC | Delta AIC | AIC weight |
| --- | --- | --- | --- | --- | --- | --- | --- | --- | --- | --- | --- | --- |
| Mod5 | x | x | x | x | x | x | x | x | - | -9778.47 | 0 | 0.7 |
| Mod4 | x | x | x | x | x | x | x | x | x | -9776.69 | 1.78 | 0.29 |

🡪 Continued on page 12.

**Recruitment of trees as response variable**

AIC selection for candidate models for the binary model component (Table S3) and the zero-truncated model component (Table S4) of the hurdle for recruitment. The models are shown in decreasing order with best fitting model at the top, and only models with ΔAICc ≤ 2 are included for better readability. However, if the model with second lowest ΔAIC had a ΔAIC ≥ 2, we show that model. Variables included in the model are noted with x, variables not included are noted with -.

**Table S3 Binary component of hurdle model, total 2 models tested**

| Model | Distance to house (600m) | log distance to road | Distance to house (600m) | Forest category | Tree species group | Autocorrelation | Edge effect | Slope | Altitude | Browsing pressure | Moose density | Tree density | Forest treatment | AIC | Delta AIC | AIC weight |
| --- | --- | --- | --- | --- | --- | --- | --- | --- | --- | --- | --- | --- | --- | --- | --- | --- |
| Mod1 | - | x | x | x | x | x | x | x | x | x | x | x | x | 21432.07 | 0 | 0.75 |
| Mod2 | x | - | x | x | x | x | - | x | x | x | x | x | x | 21434.3 | 2.24 | 0.25 |

**Table S4 Zero-truncated component of hurdle model, total 5 models tested**

| Model | Distance to house (600m) | log distance to road | Forest category | Tree species group | Autocorrelation | Edge effect | Slope | Altitude | Browsing pressure | Moose density | Tree density | Forest treatment | AIC | Delta AIC | AIC weight |
| --- | --- | --- | --- | --- | --- | --- | --- | --- | --- | --- | --- | --- | --- | --- | --- |
| Mod4 | x | x | x | x | x | x | x | x | x | - | x | - | 989246.6 | 0 | 0.41 |
| Mod3 | x | x | x | x | x | x | x | x | x | - | x | x | 989247.4 | 0.81 | 0.27 |
| Mod2 | x | x | x | x | x | x | x | x | x | x | x | - | 989248.1 | 1.55 | 0.19 |

**Comparison of R^2^ values between models with and without the explanatory variables slope, altitude and moose density**

In order to simplify the models, we tested how R^2^ values changed when dropping explanatory variables that were significant, but did not seem to effect the result of distance to infrastructure on tree recruitment much. These variables were slope, altitude and moose density. The random effect variable “municipality” acts already as a proxy of moose density on municipality level, and moose density can therefore be excluded.

The comparison shows, that by removing these variables from the best models, R2 values only change very slightly. This means, that slope, altitude and moose density indeed do not contribute much to the variation explained in the models, and hence, have little effect on the results of distance to infrastructure on tree recruitment. Considering this, we use the models without said variables.

🡪 Continued on page 13.

*Browsing pressure:*

Binary model - difference between old and new model:

Old: Marginal R^2^ / Conditional R^2^ 0.224 / 0.304

New: Marginal R^2^ / Conditional R^2^ 0.179 / 0.296

Zero-truncated model - difference between old and new model:

Old: Marginal R^2^ / Conditional R^2^ 0.543 / 0.652

New: Marginal R^2^ / Conditional R^2^ 0.539 / 0.647

*Recruitment*:

Binary - difference between old and new model:

Old: Marginal R^2^ / Conditional R^2^ 0.337 / 0.360

New: Marginal R^2^ / Conditional R^2^ 0.298 / 0.341

Zero-truncated - difference between old and new model:

Old: Marginal R^2^ / Conditional R^2^ 0.646 / 0.989

New: Marginal R^2^ / Conditional R^2^ 0.640 / 0.988

Appendix S9 Data plots of tree species groups and forest category


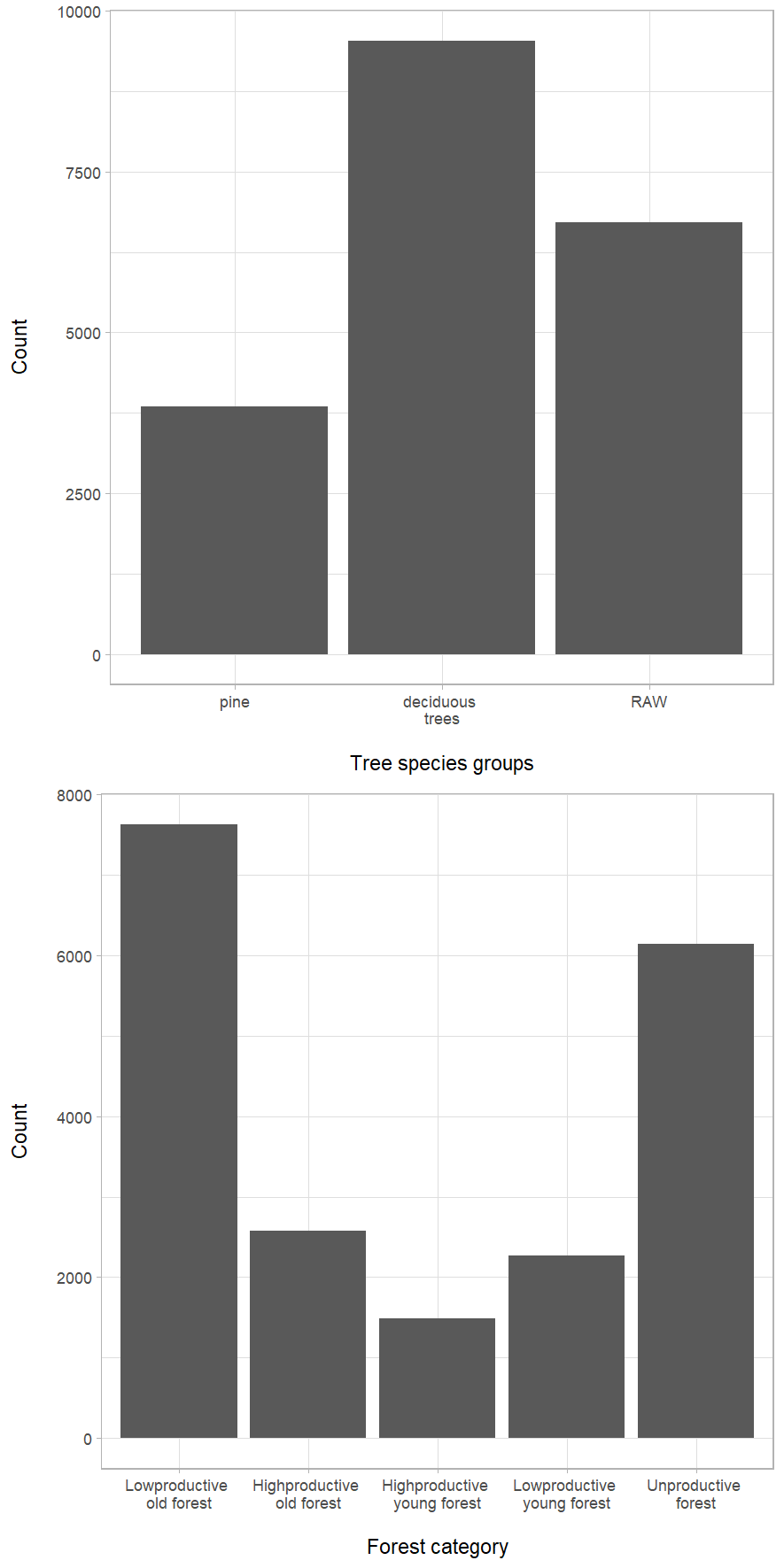


Figure S1. Data distribution of two important variables, “Tree species group” in the upper figure and “Forest category” in the bottom figure.

The most dominant tree species group was deciduous trees (other than RAW), followed by RAW (Rowan, aspen, willow) and then pine. The most dominant forest category was low-productive old forest, followed by unproductive forest.

Appendix S10 Variables changing with distance to houses and roads


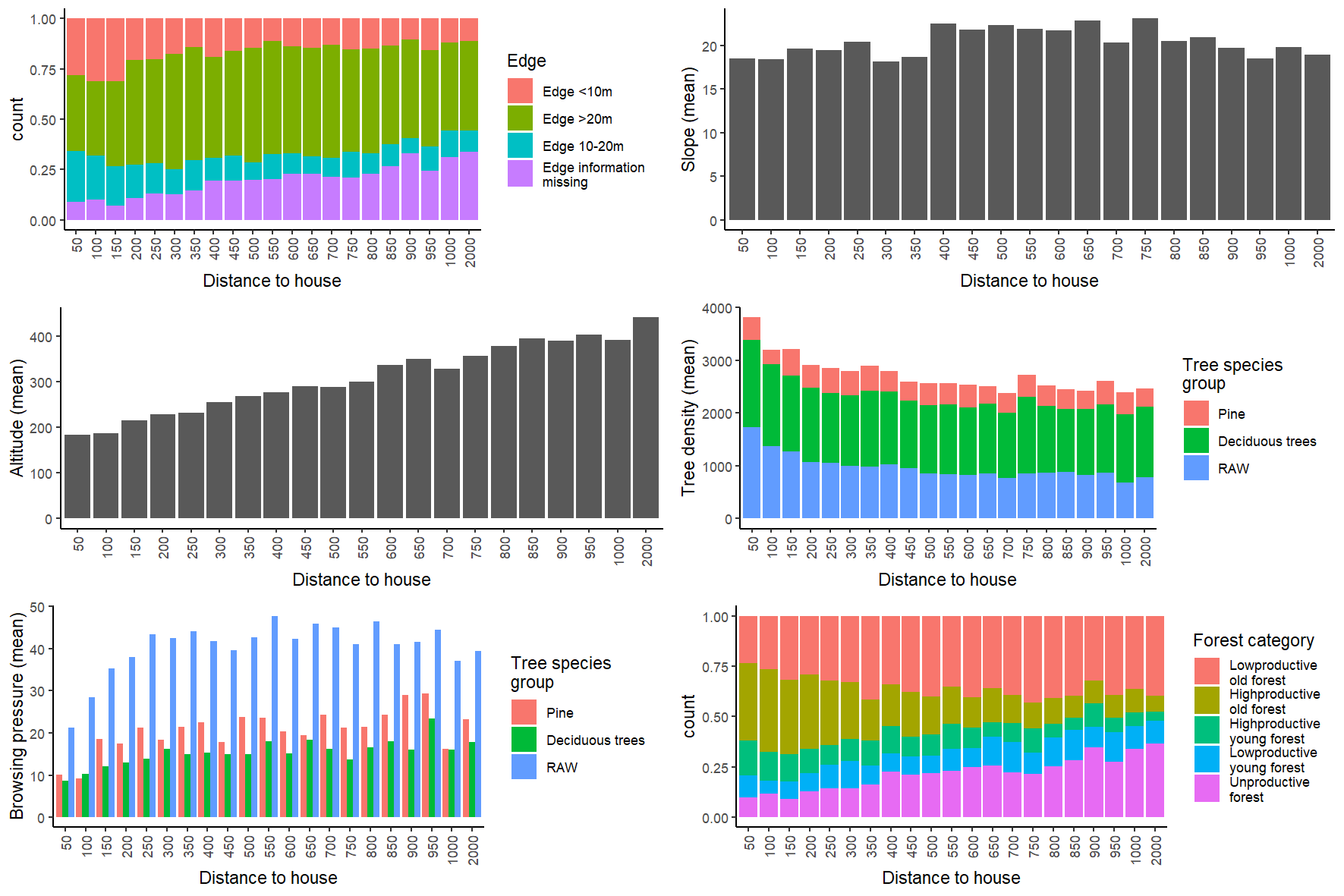


**Figure S1. Relationships between variables in the browsing pressure data.**


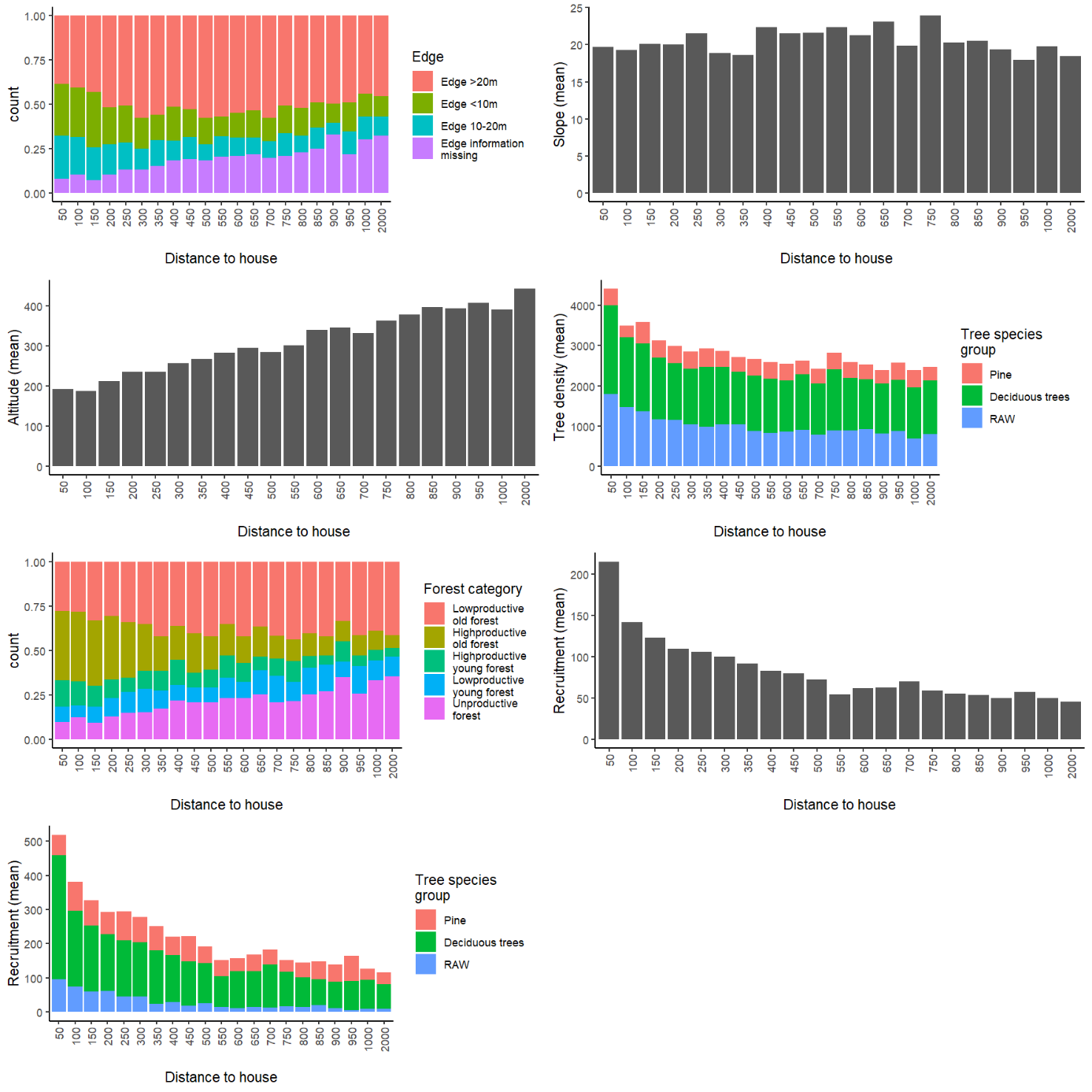


**Figure S2. Relationships between variables in the recruitment data.**

Appendix S11 Estimates and uncertainties of the final models

**Final models**

**Browsing pressure as response variable**

**Table S1 Binary component**

| **Binary model** | **Probability of browsing** | | |  | | |  |
| --- | --- | --- | --- | --- | --- | --- | --- |
| *Predictors* | *Log-Odds* | *CI* | *p* |  |  |  |  |
| (Intercept) | -1.03 | -1.40 – -0.66 | **<0.001** |  |  |  |  |
| Distance to houses (cut 200) | 0.00 | 0.00 – 0.01 | **<0.001** |  |  |  |  |
| log Distance to roads | 0.09 | 0.04 – 0.14 | **<0.001** |  |  |  |  |
| Forest category - high-productive old forest | -0.72 | -1.21 – -0.23 | **0.004** |  |  |  |  |
| Forest category - high-productive young forest | -1.67 | -2.35 – -0.99 | **<0.001** |  |  |  |  |
|  |  |  |  |  |  |  |  |
| Forest category - low-productive young forest | -0.12 | -0.77 – 0.52 | 0.711 |  |  |  |  |
|  |  |  |  |  |  |  |  |
| Forest category - unproductive forest | 0.08 | -0.43 – 0.60 | 0.748 |  |  |  |  |
| Tree species group - other deciduous trees | 0.40 | 0.30 – 0.49 | **<0.001** |  |  |  |  |
| Tree species group - RAW | 1.45 | 1.35 – 1.55 | **<0.001** |  |  |  |  |
| Tree density | 0.52 | 0.48 – 0.57 | **<0.001** |  |  |  |  |
| Edge effect <10 m | -0.25 | -0.36 – -0.14 | **<0.001** |  |  |  |  |
| Edge effect 10-20 m | -0.18 | -0.30 – -0.07 | **0.002** |  |  |  |  |
| Edge effect no information | -0.38 | -0.63 – -0.14 | **0.002** |  |  |  |  |
| Autocorrelation variable | 6.90 | 6.08 – 7.72 | **<0.001** |  |  |  |  |
| log Distance to roads*Forest category - high-productive old forest | 0.09 | 0.00 – 0.17 | **0.045** |  |  |  |  |
|  |  |  |  |  |  |  |  |
| log Distance to roads*Forest category - high-productive young forest | 0.29 | 0.18 – 0.41 | **<0.001** |  |  |  |  |
|  |  |  |  |  |  |  |  |
| log Distance to roads*Forest category - low-productive young forest | 0.03 | -0.07 – 0.14 | 0.515 |  |  |  |  |
|  |  |  |  |  |  |  |  |
| log Distance to roads*Forest category - unproductive forest | -0.04 | -0.12 – 0.03 | 0.249 |  |  |  |  |
|  |  |  |  |  |  |  |  |
| **Random Effects** | | | |  |  |  | |
| σ^2^ | 3.29 | | |  |  | |  |
| τ_00_ _Municipality_ | 0.55 | | |  |  | |  |
| ICC | 0.14 | | |  |  | |  |
| N _Municipality_ | 394 | | |  |  | |  |
| Observations | 22344 | | |  |  |  |  |
| Marginal R^2^ / Conditional R^2^ | 0.179 / 0.296 | | |  |  |  |  |

🡪 Continued on page 18.

**Table S2 Zero-truncated component, beta regression**

| **Zero-truncated model** | **Browsing pressure** | | |  |
| --- | --- | --- | --- | --- |
| *Predictors* | *Estimates* | *CI* | *p* |  |
| (Intercept) | -1.32 | -1.52 – -1.12 | **<0.001** |  |
| Distance to houses (cut 200) | 0.00 | 0.00 – 0.00 | **<0.001** |  |
| log Distance to roads | 0.04 | 0.02 – 0.07 | **0.001** |  |
| Forest category - high-productive old forest | -0.32 | -0.58 – -0.06 | **0.015** |  |
| Forest category - high-productive young forest | -0.38 | -0.72 – -0.04 | **0.03** |  |
|  |  |  |  |  |
| Forest category - low-productive young forest | 0.03 | -0.29 – 0.34 | 0.868 |  |
|  |  |  |  |  |
| Forest category - unproductive forest | -0.16 | -0.45 – 0.13 | 0.29 |  |
| Tree species group - other deciduous trees | -0.37 | -0.42 – -0.32 | **<0.001** |  |
| Tree species group - RAW | 0.6 | 0.54 – 0.65 | **<0.001** |  |
| Tree density | -0.04 | -0.06 – -0.02 | **<0.001** |  |
| Edge effect <10 m | -0.3 | -0.36 – -0.25 | **<0.001** |  |
| Edge effect 10-20 m | -0.15 | -0.21 – -0.10 | **<0.001** |  |
| Edge effect no information | -0.06 | -0.19 – 0.07 | 0.34 |  |
| Autocorrelation variable | 4.03 | 3.69 – 4.37 | **<0.001** |  |
| log Distance to roads*Forest category - high-productive old forest | 0.06 | 0.01 – 0.10 | **0.009** |  |
|  |  |  |  |  |
| log Distance to roads*Forest category - high-productive young forest | 0.10 | 0.05 – 0.16 | **<0.001** |  |
|  |  |  |  |  |
| log Distance to roads*Forest category - low-productive young forest | 0.01 | -0.04 – 0.06 | 0.575 |  |
|  |  |  |  |  |
| log Distance to roads*Forest category - unproductive forest | -0.01 | -0.05 – 0.03 | 0.525 |  |
|  |  |  |  |  |
| **Random Effects** | | | |  |
| σ^2^ | 0.21 | | |  |
| τ_00_ _Municipality_ | 0.06 | | |  |
| ICC | 0.23 | | |  |
| N _Municipality_ | 390 | | |  |
| Observations | 16663 | | |  |
| Marginal R^2^ / Conditional R^2^ | 0.539 / 0.647 | | |  |

🡪 Continued on page 19.

**Recruitment of trees as response variable**

**Table S3 Binary component**

| **Binary model** | **Probability of recruitment** | | |  |
| --- | --- | --- | --- | --- |
| *Predictors* | *Log-Odds* | *CI* | *p* |  |
| (Intercept) | 0.69 | 0.47 – 0.90 | **<0.001** |  |
| Distance to houses (cut 600) | 0.00 | -0.00 – -0.00 | **<0.001** |  |
| Forest category - high-productive old forest | 0.12 | 0.00 – 0.23 | **0.041** |  |
| Forest category - high-productive young forest | 0.55 | 0.41 – 0.69 | **<0.001** |  |
| Forest category - low-productive young forest | 0.28 | 0.16 – 0.40 | **<0.001** |  |
| Forest category - unproductive forest | 0.05 | -0.18 – 0.28 | 0.651 |  |
| log Distance to roads | -0.11 | -0.15 – -0.08 | **<0.001** |  |
| Browsing pressure | -0.01 | -0.01 – -0.00 | **<0.001** |  |
| Tree species group - other deciduous trees | 0.19 | 0.10 – 0.28 | **<0.001** |  |
| Tree species group - RAW | -1.53 | -1.64 – -1.42 | **<0.001** |  |
| Edge effect <10 m | -0.08 | -0.18 – 0.03 | 0.138 |  |
| Edge effect 10-20 m | 0.04 | -0.07 – 0.15 | 0.44 |  |
| Edge effect no information | -0.28 | -0.51 – -0.04 | **0.023** |  |
| Tree density | 0.89 | 0.84 – 0.94 | **<0.001** |  |
| Forest treatment | -0.35 | -0.52 – -0.17 | **<0.001** |  |
| Autocorrelation variable | -2.10 | -2.82 – -1.37 | **<0.001** |  |
| **Random Effects** | | | |  |
| σ^2^ | 3.29 | | |  |
| τ_00_ _Municipality_ | 0.21 | | |  |
| ICC | 0.06 | | |  |
| N _Municipality_ | 392 | | |  |
| Observations | 20520 | | |  |
| Marginal R^2^ / Conditional R^2^ | 0.298 / 0.341 | | |  |

🡪 Continued on page 20.

**Table S4 Zero-truncated component, Poisson**

| **Zero-truncated model** | **recruitment** | | |  |  |  |
| --- | --- | --- | --- | --- | --- | --- |
| *Predictors* | *Log-Mean* | *CI* | *p* |  |  |  |
| (Intercept) | 5.50 | 5.46 – 5.54 | **<0.001** |  |  |  |
| Distance to houses (cut 600) | 0.00 | -0.00 – -0.00 | **<0.001** |  |  |  |
| log Distance to roads | -0.06 | -0.06 – -0.05 | **<0.001** |  |  |  |
| Forest category - high-productive old forest | 0.08 | 0.08 – 0.09 | **<0.001** |  |  |  |
| Forest category - high-productive young forest | 0.86 | 0.85 – 0.86 | **<0.001** |  |  |  |
| Forest category - low-productive young forest | 0.55 | 0.54 – 0.55 | **<0.001** |  |  |  |
| Forest category - unproductive forest | 0.30 | 0.29 – 0.31 | **<0.001** |  |  |  |
| Tree species group - other deciduous trees | 0.01 | 0.01 – 0.02 | **<0.001** |  |  |  |
| Tree species group - RAW | -0.39 | -0.40 – -0.38 | **<0.001** |  |  |  |
| Browsing pressure | 0.00 | -0.00 – -0.00 | **<0.001** |  |  |  |
| Edge effect <10 m | 0.21 | 0.20 – 0.21 | **<0.001** |  |  |  |
| Edge effect 10-20 m | 0.10 | 0.10 – 0.11 | **<0.001** |  |  |  |
| Edge effect no information | -0.33 | -0.34 – -0.32 | **<0.001** |  |  |  |
| Tree density | 0.37 | 0.37 – 0.37 | **<0.001** |  |  |  |
| Autocorrelation variable | -0.65 | -0.70 – -0.61 | **<0.001** |  |  |  |
| **Random Effects** | | | |  |  |  |
| σ^2^ | 0.01 | | |  |  |  |
| τ_00_ _Municipality_ | 0.18 | | |  |  |  |
| ICC | 0.97 | | |  |  |  |
| N _Municipality_ | 380 | | |  |  |  |
| Observations | 7464 | | |  |  |  |
| Marginal R^2^ / Conditional R^2^ | 0.640 / 0.988 | | |  |  |  |

Appendix S12 Figures of model results of the highest ranked models

**Browsing pressure hurdle**


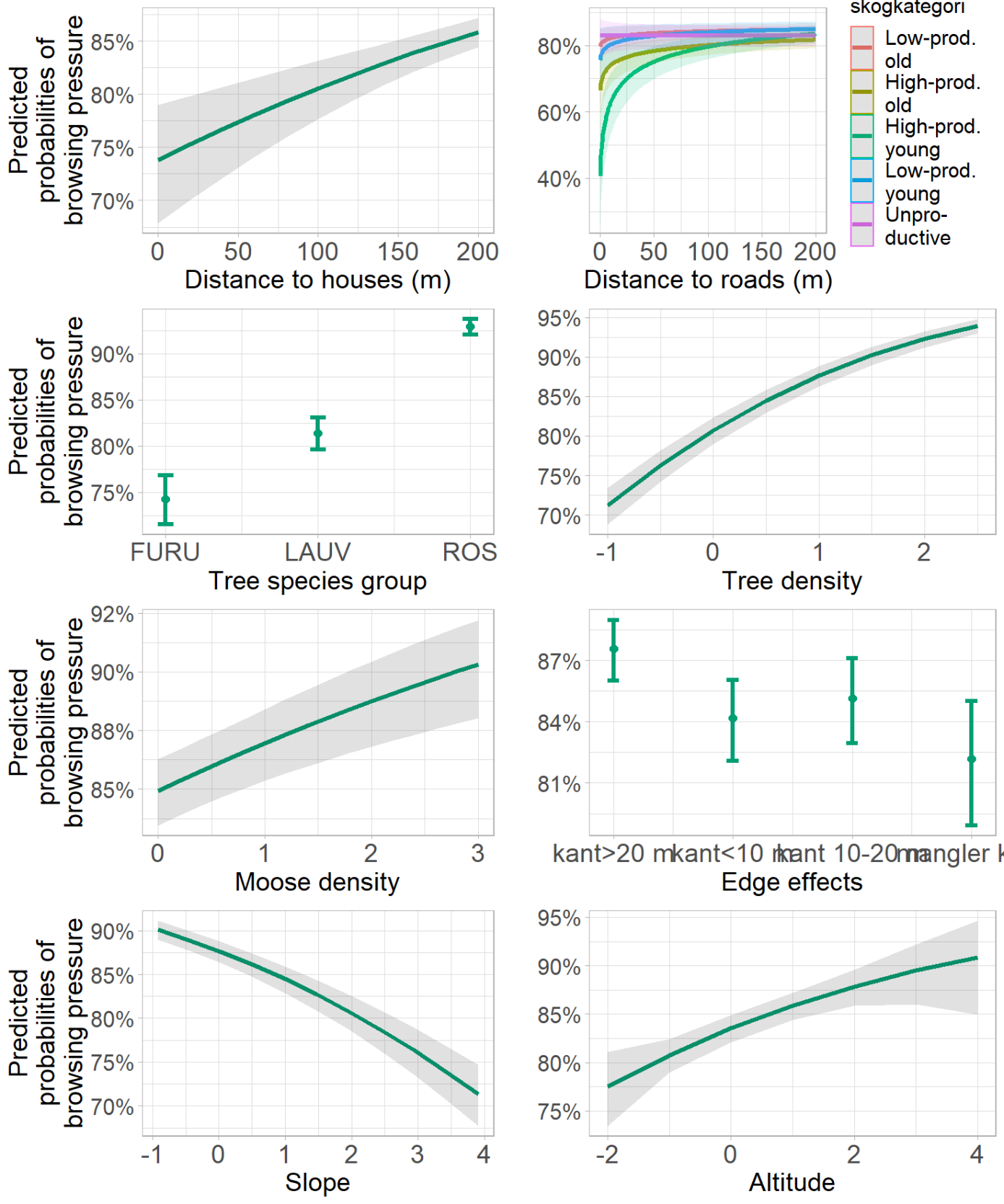


Figure S1 Effect-plots of variables affecting the predicted probability of browsing (0/1).

(a) Distance to house, (b) interaction distance to road with forest category (Low-productive old forest, high-productive old forest, high-productive young forest, low-productive young forest, unproductive forest), (c) tree species group, (d) tree density, (e) moose density, and (f) edge effects. Tree density is scaled.


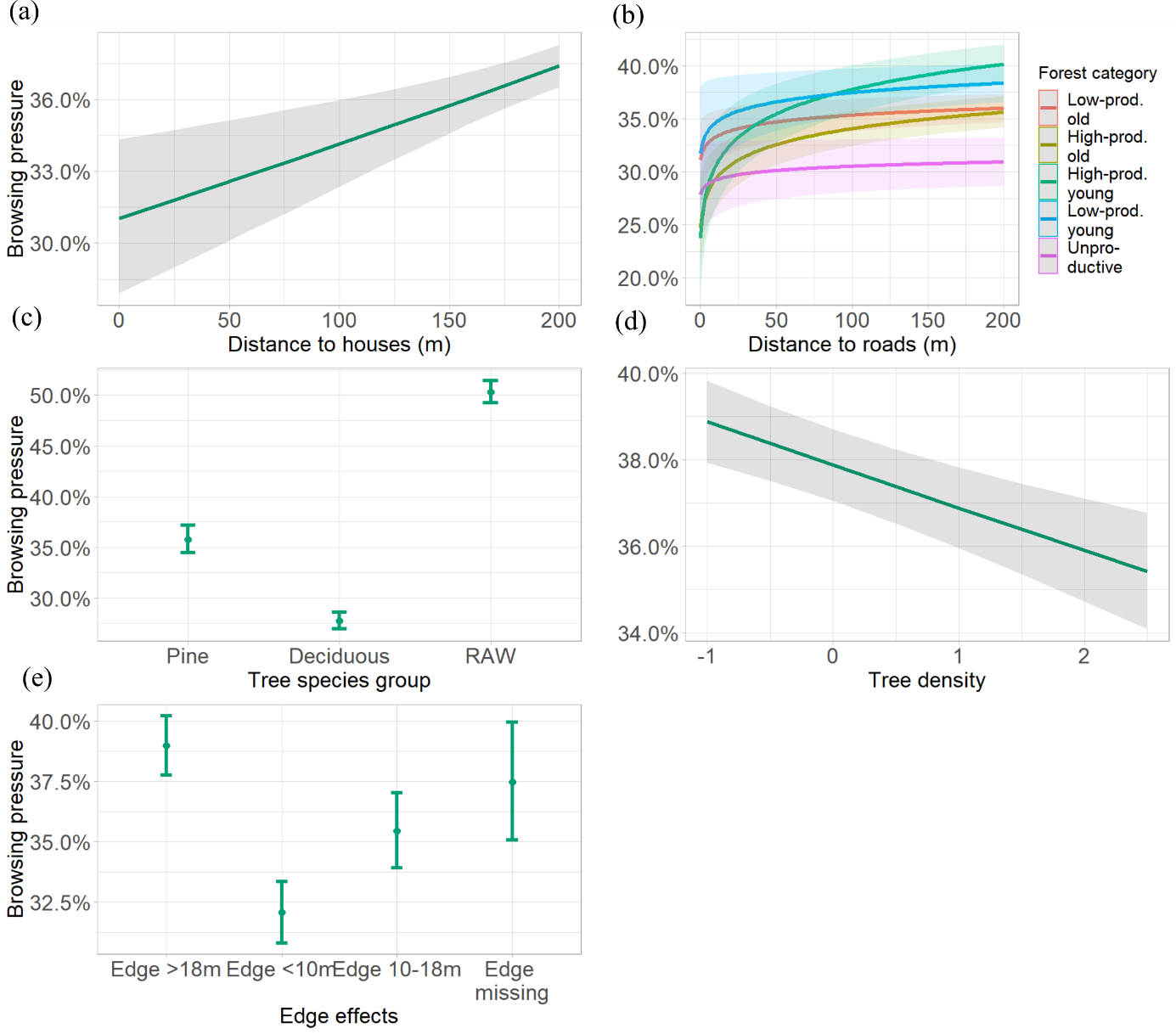


Figure S2 Effect-plots of variables affecting browsing pressure (Zero-truncated beta regression). (a) Distance to house, (b) interaction distance to road with forest category (Low-productive old forest, high-productive old forest, high-productive young forest, low-productive young forest, unproductive forest), (c) tree species group, (d) tree density, and (e) edge effects. Tree density is scaled.


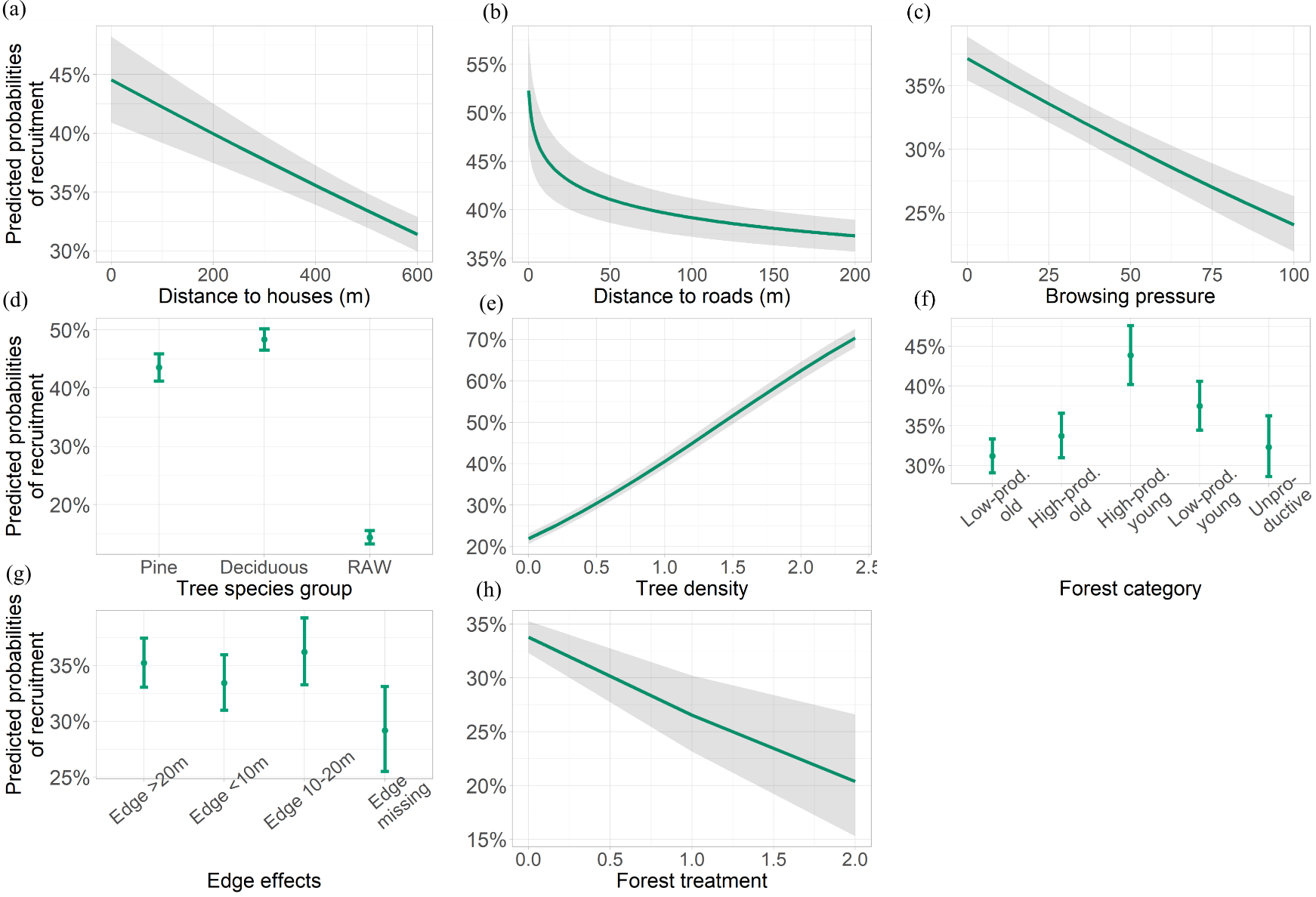
Figure S3 Effect-plots of variables affecting the predicted probability of recruitment (0/1).

(a) Distance to house, (b) distance to road, (c) browsing pressure, (d) tree species group, (e) tree density, (f) forest category (Low-productive old forest, high-productive old forest, high-productive young forest, low-productive young forest, unproductive forest), (g) edge effects, and (h) forest treatment. Tree density is scaled.


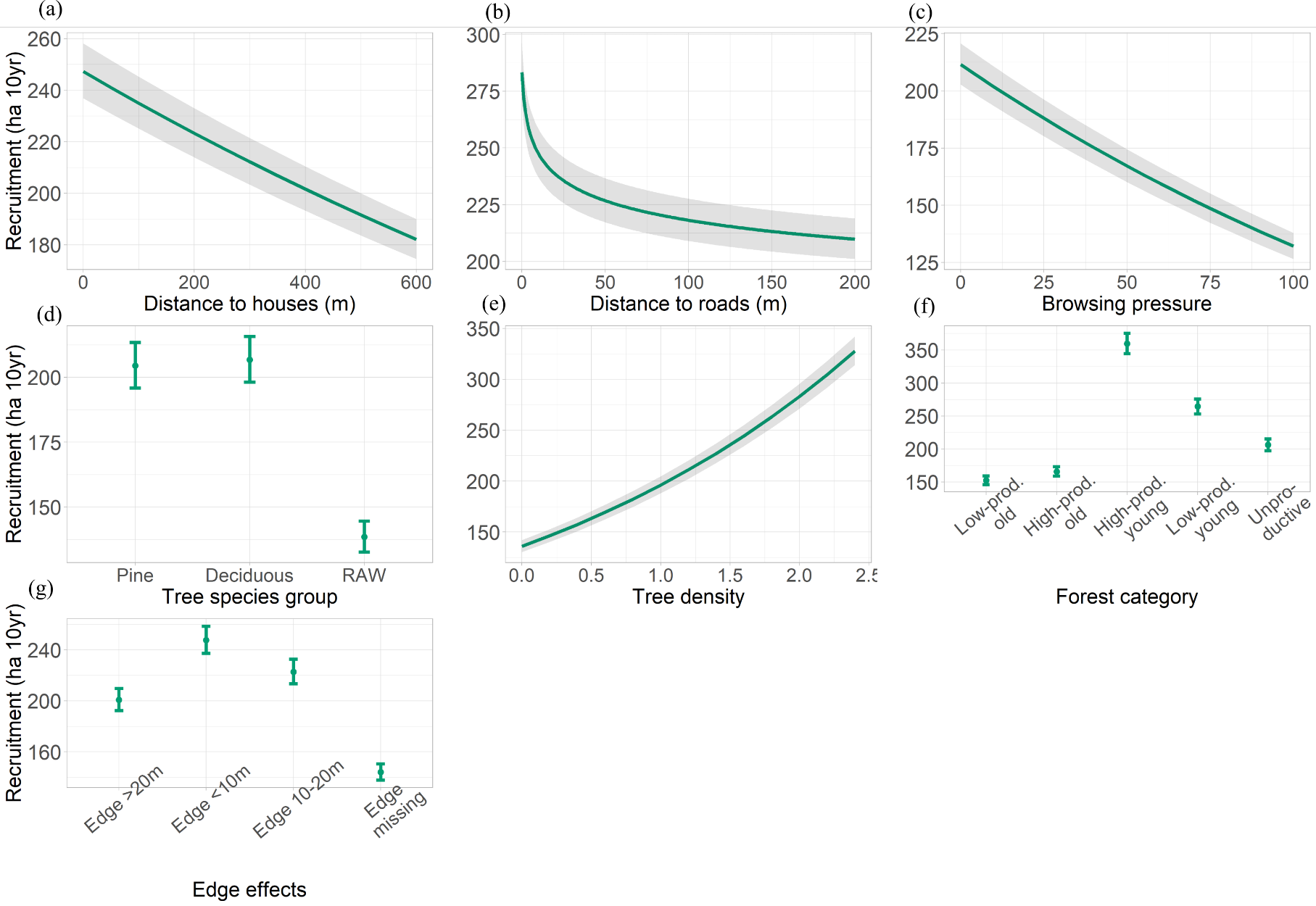
Figure S4 Effect-plots of variables affecting recruitment (Zero-truncated Poisson).

(a) Distance to house, (b) distance to road, (c) browsing pressure, (d) tree species group, (e) tree density, (f) forest category (Low-productive old forest, high-productive old forest, high-productive young forest, low-productive young forest, unproductive forest), and (g) edge effects. Tree density is scaled.

Appendix S13 Comparison of recruitment models with and without browsing pressure

**Table S1. Summary recruitment model binary component, with browsing pressure**

|  | Estimate | Std. Error | z value | Pr(>\|z\|) |  |
| --- | --- | --- | --- | --- | --- |
| (Intercept) | 0.64614 | 0.12572 | 5.139 | 2.76E-07 | *** |
| Distance to houses (cut 600) | -0.15315 | 0.02115 | -7.242 | 4.43E-13 | *** |
| Forest category - high-productive old forest | 0.11364 | 0.05662 | 2.007 | 0.0447 | * |
| Forest category - high-productive young forest | 0.54412 | 0.07158 | 7.601 | 2.93E-14 | *** |
| Forest category - low-productive young forest | 0.27916 | 0.05904 | 4.728 | 2.26E-06 | *** |
| Forest category - unproductive forest | 0.05271 | 0.11697 | 0.451 | 0.6522 |  |
| log Distance to roads | -0.11543 | 0.01785 | -6.467 | 1.00E-10 | *** |
| Browsing pressure | -0.19247 | 0.02089 | -9.212 | < 2e-16 | *** |
| Tree species group - other deciduous trees | 0.19436 | 0.04556 | 4.266 | 1.99E-05 | *** |
| Tree species group - RAW | -1.52604 | 0.05619 | -27.16 | < 2e-16 | *** |
| Edge effect <10 m | -0.07982 | 0.05348 | -1.493 | 0.1355 |  |
| Edge effect 10-20 m | 0.04168 | 0.0559 | 0.746 | 0.4559 |  |
| Edge effect no information | -0.27697 | 0.12132 | -2.283 | 0.0224 | * |
| Tree density | 0.67118 | 0.01913 | 35.078 | < 2e-16 | *** |
| Forest treatment | -0.34613 | 0.08847 | -3.912 | 9.14E-05 | *** |
| Autocorrelation variable | -2.09082 | 0.36982 | -5.654 | 1.57E-08 | *** |

**Table S2. Summary recruitment model binary component, without browsing pressure**

|  | Estimate | Std. Error | z value | Pr(>\|z\|) |  |
| --- | --- | --- | --- | --- | --- |
| (Intercept) | 0.75812 | 0.12511 | 6.06 | 1.37E-09 | *** |
| Distance to houses (cut 600) | -0.15732 | 0.0211 | -7.454 | 9.03E-14 | *** |
| Forest category - high-productive old forest | 0.11941 | 0.05649 | 2.114 | 0.034524 | * |
| Forest category - high-productive young forest | 0.50254 | 0.07105 | 7.073 | 1.52E-12 | *** |
| Forest category - low-productive young forest | 0.25752 | 0.05879 | 4.38 | 1.19E-05 | *** |
| Forest category - unproductive forest | 0.07842 | 0.11718 | 0.669 | 0.503344 |  |
| log Distance to roads | -0.12403 | 0.01782 | -6.961 | 3.38E-12 | *** |
|  |  |  |  |  |  |
| Tree species group - other deciduous trees | 0.22265 | 0.04531 | 4.914 | 8.93E-07 | *** |
| Tree species group - RAW | -1.64885 | 0.05475 | -30.115 | < 2e-16 | *** |
| Edge effect <10 m | -0.0343 | 0.05318 | -0.645 | 0.518896 |  |
| Edge effect 10-20 m | 0.06916 | 0.0557 | 1.242 | 0.214365 |  |
| Edge effect no information | -0.25099 | 0.12151 | -2.066 | 0.038863 | * |
| Tree density | 0.66042 | 0.01902 | 34.725 | < 2e-16 | *** |
| Forest treatment | -0.34148 | 0.0879 | -3.885 | 0.000102 | *** |
| Autocorrelation variable | -2.78024 | 0.36297 | -7.66 | 1.86E-14 | *** |

🡪 Continued on page 26.

**Table S3. Summary recruitment model zero-truncated component, with browsing pressure**

|  | Estimate | Std. Error | z value |  | Pr(>\|z\|) |  |
| --- | --- | --- | --- | --- | --- | --- |
| (Intercept) | 5.172666 | 0.022619 | 228.688 | < | 2E-16 | *** |
| Distance to houses (cut 600) | -0.09082 | 0.001143 | -79.476 | < | 2E-16 | *** |
| log Distance to roads | -0.05668 | 0.000888 | -63.829 | < | 2E-16 | *** |
| Forest category - high-productive old forest | 0.08246 | 0.003262 | 25.28 | < | 2E-16 | *** |
| Forest category - high-productive young forest | 0.856816 | 0.003116 | 274.986 | < | 2E-16 | *** |
| Forest category - low-productive young forest | 0.548953 | 0.002912 | 188.494 | < | 2E-16 | *** |
| Forest category - unproductive forest | 0.300102 | 0.005808 | 51.667 | < | 2E-16 | *** |
| Tree species group - other deciduous trees | 0.01096 | 0.003034 | 3.613 | < | 0.000303 | *** |
| Tree species group - RAW | -0.38941 | 0.004029 | -96.651 | < | 2E-16 | *** |
| Browsing pressure | -0.11908 | 0.001155 | -103.103 | < | 2E-16 | *** |
| Edge effect <10 m | 0.209609 | 0.002487 | 84.289 | < | 2E-16 | *** |
| Edge effect 10-20 m | 0.103828 | 0.002948 | 35.217 | < | 2E-16 | *** |
| Edge effect no information | -0.33235 | 0.006131 | -54.207 | < | 2E-16 | *** |
| Tree density | 0.366849 | 0.000933 | 393.388 | < | 2E-16 | *** |
| Autocorrelation variable | -0.65385 | 0.022489 | -29.074 | < | 2E-16 | *** |

**Table S4. Summary recruitment model zero-truncated component, without browsing pressure**

|  | Estimate | Std. Error | z value | Pr(>\|z\|) |  |
| --- | --- | --- | --- | --- | --- |
| (Intercept) | 5.23647 | 0.022605 | 231.655 | < 2e-16 | *** |
| Distance to houses (cut 600) | -0.09972 | 0.001138 | -87.626 | < 2e-16 | *** |
| log Distance to roads | -0.06326 | 0.000886 | -71.4 | < 2e-16 | *** |
| Forest category - high-productive old forest | 0.086991 | 0.003261 | 26.676 | < 2e-16 | *** |
| Forest category - high-productive young forest | 0.845975 | 0.003114 | 271.653 | < 2e-16 | *** |
| Forest category - low-productive young forest | 0.541485 | 0.00291 | 186.061 | < 2e-16 | *** |
| Forest category - unproductive forest | 0.316823 | 0.005804 | 54.584 | < 2e-16 | *** |
| Tree species group - other deciduous trees | 0.018927 | 0.003029 | 6.248 | 4.15E-10 | *** |
| Tree species group - RAW | -0.49803 | 0.0039 | -127.708 | < 2e-16 | *** |
|  |  |  |  |  |  |
| Edge effect <10 m | 0.234798 | 0.002476 | 94.835 | < 2e-16 | *** |
| Edge effect 10-20 m | 0.118563 | 0.002945 | 40.26 | < 2e-16 | *** |
| Edge effect no information | -0.32134 | 0.00613 | -52.423 | < 2e-16 | *** |
| Tree density | 0.362261 | 0.000931 | 389.208 | < 2e-16 | *** |
| Autocorrelation variable | -1.01268 | 0.022296 | -45.419 | < 2e-16 | *** |

Appendix S14 Estimates and uncertainties of an additional model with tree density as response

Table S1. Table of model estimates for model with tree density as response.

|  | **log Tree density** | | |
| --- | --- | --- | --- |
| *Predictors* | *Estimates* | *CI* | *p* |
| (Intercept) | 5.12 | 5.05 – 5.18 | **<0.001** |
| Distance to houses (cut 600) | -0.1 | -0.11 – -0.08 | **<0.001** |
| High productive old forest | -0.03 | -0.09 – 0.02 | 0.229 |
| High productive young forest | 0.56 | 0.49 – 0.63 | **<0.001** |
| Low productive young forest | 0.47 | 0.41 – 0.53 | **<0.001** |
| Unproductive forest | 0.23 | 0.12 – 0.34 | **<0.001** |
| Edge <10m | 0.34 | 0.28 – 0.39 | **<0.001** |
| Edge 10-20m | 0.12 | 0.07 – 0.17 | **<0.001** |
| Edge no information | 0.03 | -0.09 – 0.14 | 0.637 |
| Slope | 0.05 | 0.03 – 0.07 | **<0.001** |
| Tree species group - deciduous | 1.32 | 1.27 – 1.36 | **<0.001** |
| Tree species group - RAW | 0.63 | 0.58 – 0.68 | **<0.001** |
| Browsing pressure | 0.00 | 0.00 – 0.00 | **<0.001** |
| Autocorrelation | -1.16 | -1.52 – -0.81 | **<0.001** |
| **Random Effects** | | | |
| σ^2^ | 1.34 | | |
| τ_00_ _MUNICIPALITY_ | 0.12 | | |
| ICC | 0.08 | | |
| N _MUNICIPALITY_ | 392 | | |
| Observations | 20534 | | |
| Marginal R^2^ / Conditional R^2^ | 0.172 / 0.239 | | |
| AIC | 64835.47 | | |

🡪 Continued on page 28.


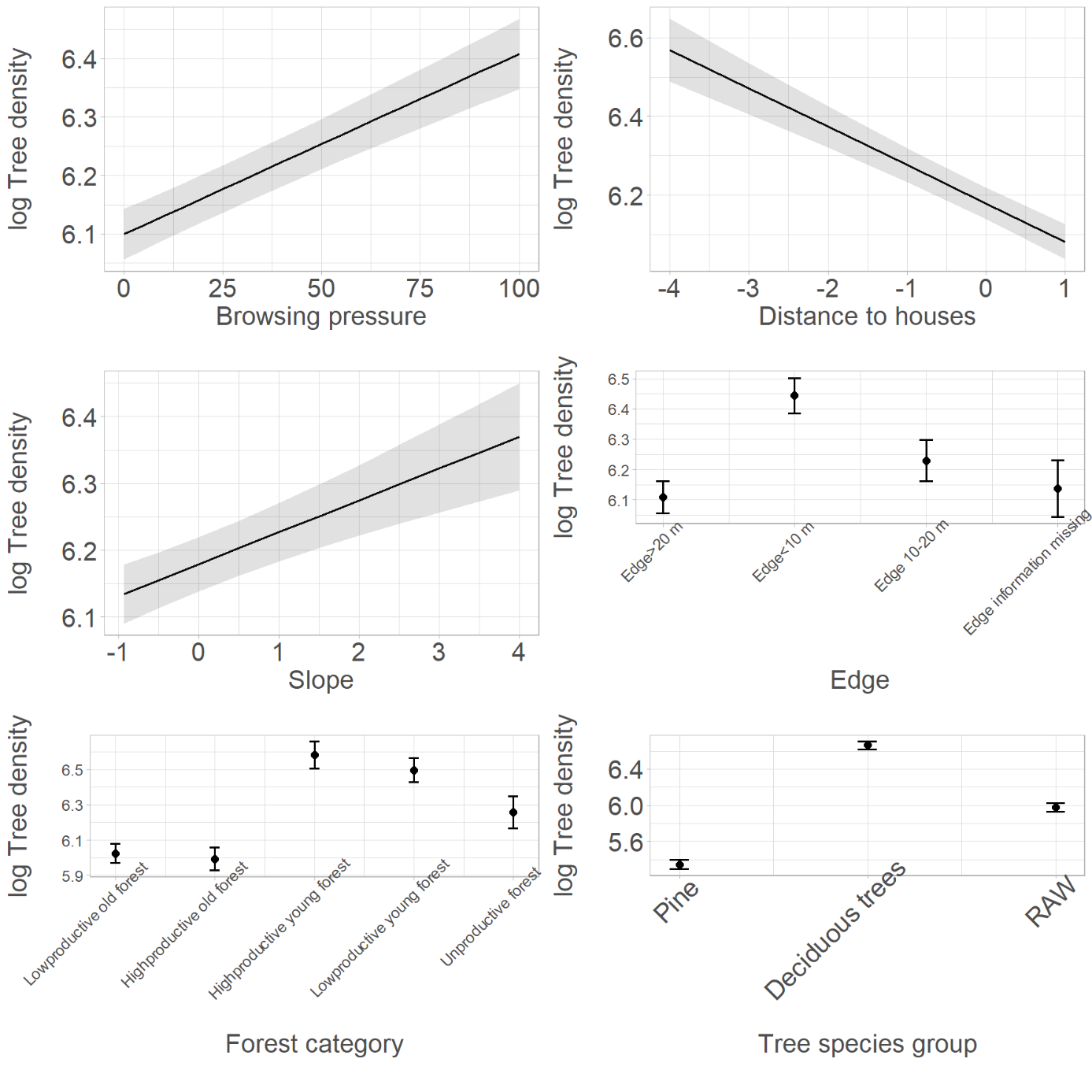


Figure S1. Effect-plots of variables affecting the predicted tree density. Distance to house is scaled and centered and ranges from 6 to 600 m, slope is scaled and centered as well and ranges from 1 to 100.
